# Dose‐dependent effect of megestrol acetate supplementation in cancer patients with anorexia–cachexia syndrome: A meta‐analysis

**DOI:** 10.1002/jcsm.13500

**Published:** 2024-06-20

**Authors:** Sepide Talebi, Sheida Zeraattalab‐Motlagh, Maryam Barkhordar, Mohammad Vaezi, Seyed Mojtaba Ghoreishy, Abed Ghavami, Yasaman Hosseini, Nikolaj Travica, Hamed Mohammadi

**Affiliations:** ^1^ Students' Scientific Research Center (SSRC) Tehran University of Medical Sciences Tehran Iran; ^2^ Department of Clinical Nutrition, School of Nutritional Sciences and Dietetics Tehran University of Medical Sciences Tehran Iran; ^3^ Department of Health and Human Performance University of Houston Houston TX USA; ^4^ Cell Therapy and Hematopoietic Stem Cell Transplantation Research Center Tehran University of Medical Sciences Tehran Iran; ^5^ Hematology, Oncology and Stem Cell Transplantation Research Center Tehran University of Medical Sciences Tehran Iran; ^6^ Research Institute for Oncology, Hematology and Cell Therapy Tehran University of Medical Sciences Tehran Iran; ^7^ Department of Nutrition, School of Public Health Iran University of Medical Sciences Tehran Iran; ^8^ Student Research Committee, School of Public Health Iran University of Medical Sciences Tehran Iran; ^9^ Department of Clinical Nutrition, School of Nutrition and Food Science Isfahan University of Medical Sciences Isfahan Iran; ^10^ IMPACT—Institute for Mental and Physical Health and Clinical Translation, Food and Mood Centre, School of Medicine, Barwon Health Deakin University Geelong Australia

**Keywords:** megestrol acetate, cancer, cachexia, anorexia, appetite

## Abstract

There is inconsistent evidence relating to the effects of megestrol acetate (MA) supplementation on cancer patients suffering from anorexia–cachexia syndrome. This review aimed to examine the dose–response effect of MA supplementation in patients with cancer‐associated anorexia/cachexia. Relevant keywords were searched in PubMed, Scopus and ISI Web of Science from inception to June 2023 for randomized controlled trials (RCTs) examining the effect of MA on pathologies in patients with cancer‐associated cachexia. Our primary outcomes were changes in body weight and appetite. However, fatigue and quality of life were secondary outcomes. The mean difference (MD) and 95% confidence interval (95% CI) were estimated using the random‐effects method. Thirteen trials comprising 1229 participants (mean age 60 years) were identified. The results of our highest versus lowest analysis revealed that MA supplementation was not associated with any increase in body weight (MD: 0.64 kg, 95% CI [−0.11, 1.38], *P* = 0.093, *I* ^2^ = 69.1%; GRADE = very low certainty). Twelve trials, including 14 effect sizes derived from 1369 patients (intervention = 689, control = 680), provided data on the effect of MA on body weight. Subgroup analyses showed a significant increase in body weight following short‐term intervention (≤8 weeks) and a combination of radiation/chemotherapy as concurrent treatment. A linear dose–response meta‐analysis indicated that each 200 mg/day increment in MA consumption had a significant increase in weight gain (MD: 0.44; 95% CI [0.13, 0.74], *P* = 0.005; *I* ^2^ = 97.1%); however, the magnitude of the effect was small. MA administration significantly affected the quality of life based on pooled effect sizes (MD: 1.15, 95% CI [0.76, 1.54], *P* < 0.001, *I* ^2^ = 0%; *n* = 2 RCTs including 176 patients; GRADE = very low certainty). However, no significant effect of MA supplementation was observed on appetite (MD: 0.29, 95% CI [−0.05, 0.64], *P* = 0.096, *I* ^2^ = 18.3%; *n* = 3 RCTs including 163 patients; GRADE = very low certainty) and fatigue (MD: 0.14, 95% CI [−0.09, 0.36], *P* = 0.236, *I* ^2^ = 0%; *n* = 2 RCTs including 300 patients; GRADE = very low certainty). With very low certainty of the evidence, MA supplementation may not lead to a significantly increased weight gain and other outcomes.

## Introduction

The frequently related cause of malnourishment in cancer patients is cachexia.^1^ It is a condition that adversely affects quality of life,^2^ contributes to progressive dysfunction, increases the risk of mortality and morbidity^3^ and increases post‐operative difficulties.^4^ The criteria used for recognition of cancer cachexia is defined as weight reduction ≥5% or ≥2% based on a patient's recent body mass index (BMI) (<20 kg.m^−2^) or decreases in skeletal muscle mass (sarcopenia).^5^ In 2014, cachexia was present in 50–80% of patients with cancer, leading to a mortality rate of 80% and cancer fatality of 20%.^6^

The underlying mechanism causing cancer cachexia is not well understood; inflammatory cytokines, including tumour necrosis factor‐alpha and interleukin‐6, potentially have a role.^7^ These patients also experience higher levels of ghrelin hormone.^8^ Hence, implementing dietary counselling, nutritional therapies, and support are crucial and ratified by clinical practice guidelines.^9^ Nevertheless, approaches might be limited as patients with cancer cachexia are mostly refractory to nutritional support.^10^

Managing cancer‐associated cachexia is challenging, and various pharmacological therapies with variable efficacy and tolerability on clinical and quality of life outcomes have been examined.^11^, ^12^, ^13^ For instance, megestrol acetate (MA), a synthetic hormone (progestin), is administered to improve body weight and appetite in cancer‐related cachexia.^12^, ^14^ Based on clinical studies, supplementation with MA has shown the ability to improve weight gain and appetite^12^, ^14^, ^15^ and reduce inflammatory cytokines.^14^

However, recent systematic reviews have indicated inconclusive results. Ruiz‐García et al. did not show any favourable changes in the efficacy of MA, although this study included patients with non‐specific pathology‐related cachexia/anorexia.^16^ A recent meta‐analysis of 23 trials indicated that MA did not significantly affect weight gain in cancer‐associated anorexia/cachexia.^17^ Since then, more recent trials have been published.^18^, ^19^, ^20^, ^21^, ^22^, ^23^, ^24^, ^25^ Moreover, no systematic review and meta‐analysis have examined the pattern of the relationship between MA supplementation and pathologies in cancer patients suffering from anorexia–cachexia syndrome, with the use of a dose–response meta‐analysis that applies fractional polynomial modelling. In addition, uncertainty remains about the efficacy of MA in cancer cachexia. As a result, we conducted a comprehensive systematic review and meta‐analysis of randomized controlled trials (RCTs) to clarify the dose–response effect of MA on anorexia–cachexia syndrome in patients with cancer.

## Methods

We adhered to the Preferred Reporting Items for Systematic Reviews and Meta‐Analysis (PRISMA) guidelines^26^ and the Grading of Recommendations Assessment, Development and Evaluation (GRADE) framework to conduct our meta‐analysis.^27^ Our study protocol was registered at PROSPERO (ID: CRD42022358849). However, we have made changes to our systematic review protocol to broaden the search profile and focus on patient‐relevant outcomes and updated PROSPERO.

### Search strategy

We conducted a systematic literature search of appropriate studies from inception up to June 2023 using PubMed, Scopus, ISI Web of Science data sources, and clinical trials registries, such as ISRCTN registry (www.isrctn.com/editAdvancedSearch), ClinicalTrials.gov (www.clinicaltrials.gov), and the World Health Organization (WHO) International Clinical Trials Registry Platform (ICTRP) (www.who.int/ictrp/search/en). Two core keywords were utilized to examine the databases, including ‘Megestrol acetate’ and ‘Cancer’ in addition to their MeSH terms combined with related terms and the term ‘clinical trial’ to ascertain the relevant studies. Moreover, a second manual search on the references of retrieved articles was undertaken. *Tables* *S1* and *S2* include details of the search strategy, keywords used for each database and the primary result of the search and excluded studies. Two reviewers (S. T. and S. Z. M.) separately searched the electronic databases based on PICOS (participants: cancer patients; intervention: MA; comparison: placebo or other control groups; outcomes: body weight, appetite, fatigue and European Organization for Research and Treatment of Cancer Quality of Life Questionnaire C30 (EORTC‐QLQ‐C30; and study design: RCT). Any indecision was resolved by consensus with a third author (H. M.).

### Study selection

Following the removal of study duplicates, the remaining articles have their titles, abstracts, and full texts screened individually by two reviewers. Studies that satisfied the subsequent criteria were eligible for inclusion: (a) RCTs with either parallel or cross‐over study designs; (b) studies conducted with cancer patients aged >18 years; (c) those assessing the impact of MA supplementation on body weight, appetite, fatigue and quality of life, and (d) those providing adequate details on the value of baseline and end of trial outcomes (or reported changes in outcomes) for both MA and control groups.

Studies were omitted if they lacked a suitable control, and MA was administrated as a complex with other drugs or active substances. In the case that the studies reported the change of outcomes in multiple doses of MA in comparison to the placebo/control group, data from both doses were selected for analysis.

### Data extraction

Two authors (Y. H. and S. T.) independently performed the data extraction, which included publication year, country of the study, study design and blinding, underlying malignancy, sex, mean age of participants, concurrent treatment such as chemotherapy, radiotherapy, and other palliative care, sample size, type and dose of supplement and placebo, intervention duration. Consequently, the data were cross‐referenced, and discrepancies were resolved through discussion with a third author (H. M.).

### Risk of bias assessment

Two reviewers (Y. H. and S. T.) administered the Cochrane Collaboration Risk of Bias (RoB) tool^28^ to assess the risk of bias based on the following criteria: (a) random sequence generation adequacy (selection bias); (b) allocation concealment (selection bias); (c) blinding of participants and investigators (performance bias); (d) the explanation of failures and inadequate outcome data (attrition bias), (e) selective reporting of the outcomes (reporting bias) and (f) other probable sources of bias. Each domain was rated as either ‘low’, ‘high’ and ‘unclear’ risk of bias per Cochrane Handbook. Any variance in the risk of bias assessment was resolved between reviewers (*Table* *S3*).

### Statistical analysis

All statistical analyses were performed using STATA software version 17 (STATA Corp, College Station, TX, USA). Data from main outcomes such as weight, appetite, fatigue and EORTC QLQ‐C30 included mean change and standard deviation (*SD*) to assess the overall effect size. Weighted (WMD) or standardized (SMD) mean differences with 95% confidence interval (CI) were used to determine MA effects on weight, appetite, fatigue and the EORTC QLQ‐C30.

If studies failed to report data directly, the mean change was calculated by subtracting baseline measures from values post‐intervention. The standard deviation for the calculated net change was computed by using the formula proposed by Follmann et al.^29^ where: root square of (*SD* baseline^2^ + *SD* final^2^) − (2 × *R* × *SD* baseline × *SD* final); where *R* represents a correlation coefficient of 0.5.^30^ Given that pre‐test to post‐test *R* was not reported in RCTs, an *R*‐value of 0.5 was utilized throughout this meta‐analysis.^31^ Moreover, we conducted sensitivity analyses using different correlation values (0.25 and 0.75), reporting and interpreting results from meta‐analyses (*Table* *S4*). Due to the observed variation in study treatments and protocol, a random‐effects model was implemented to estimate mean difference (MD) and 95% CIs.^31^ The between‐studies heterogeneity was evaluated by the I‐squared (*I* ^2^) index, with an *I* ^2^ value of <50% considered as ‘moderate’ heterogeneity, *I* ^2^ ≥ 50% and ≤75% considered as ‘substantial’ heterogeneity and ‘considerable’ heterogeneity defined by *I* ^2^ ≥ 75%.^31^ Subgroup analysis was performed based on the dose (≤320 g/day as a low dose and >320 g/day as a high dose) and duration (≤8 weeks as short term and >8 weeks as long term) of MA supplementation, type of concurrent treatment (chemotherapy, radiotherapy plus chemotherapy) and type of control group (placebo or medication as placebo group) to explore the source of heterogeneity. Additionally, we conducted a dose–response meta‐analysis to clarify the pattern of the effect of different doses of MA on body weight and appetite.^32^

Sensitivity analysis was undertaken to observe the degree to which results may be influenced by a particular study or a group of studies with a high risk of bias.^33^ Publication bias was assessed by the Begg—adjusted rank correlation test and Egger's regression asymmetry test.^34^ The trim and fill test was undertaken to establish the possible effect of the lack of specific studies if there was any evidence of publication bias. A *P* value <0.05 was regarded as statistically significant.

### Grading the evidence

The GRADE^35^ method was employed to evaluate the certainty of evidence. The quality of the assessed evidence was rated as high, moderate, low or very low. High grades are indicative of high confidence that the actual effect is commensurate with the estimated effect. Moderate grades suggest that the actual effect is likely to be close to the estimate of the effect; however, there exists a small possibility of substantial differences. A low grade suggests a greater likelihood that the true effect may be substantially different from the estimate of the effect, and very low grades suggest the true effect is likely different from the estimated effect. Further, RCTs with an initial high‐quality evidence evaluation were downgraded based on study limitations, including the risk of bias, inconsistency (substantial unexplained heterogeneity, *I* ^2^ > 50%; *P* < 0.05) and imprecision (95% CI for effect estimates that are wide or did not cross the minimal clinically important difference [MCID] threshold for any outcome, which was regarded as clinically important difference). Indirectness of outcomes (primary outcomes presented are surrogate rather than patient‐important outcomes)^35^ and other considerations (such as publication bias and dose–response gradient usage) further affected the grading of evidence. The MCID for our outcomes were as follows: weight (2.5 kg), appetite (1.6), fatigue (0.9), and EORTC QLQ‐C30 (at least 3‐point increment on QLQ‐C30 domains).^36^, ^37^, ^38^

## Results

### Study selection

**Figure 1:**
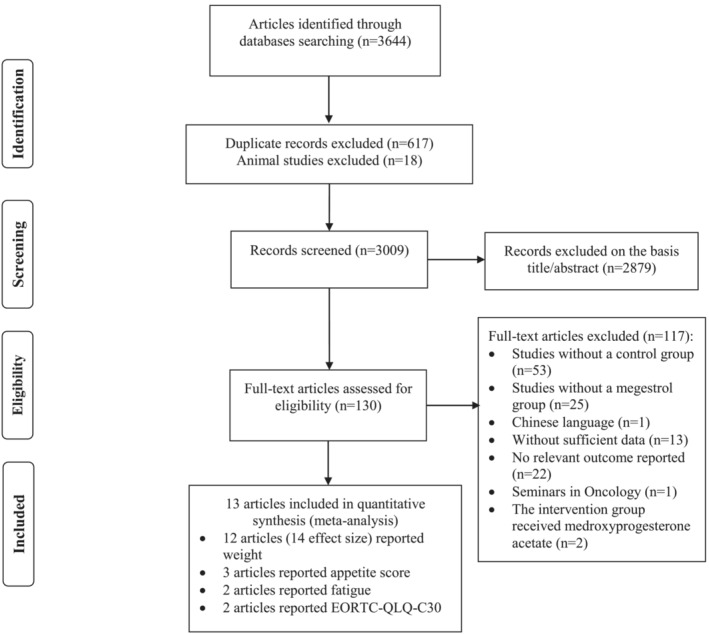
Flow diagram of study selection.

A total of 3644 records were initially retrieved, of whom 617 duplicate publications and 18 animal studies were removed. After screening based on titles/abstracts, 2879 articles were removed. We included 130 articles for further examination of full texts. Of these articles, 117 studies were excluded due to the following reasons: lacking a control group (*n* = 53), studies without a MA group (*n* = 25), without sufficient data for outcomes (baseline or final assessment) (*n* = 13), no relevant outcome reported (*n* = 22), the intervention group received medroxyprogesterone acetate (*n* = 2) and one study was as a seminar in oncology. Thirteen articles were included in the final quantitative analysis.^18^, ^19^, ^20^, ^21^, ^22^, ^23^, ^24^, ^25^, ^39^, ^40^, ^41^, ^42^, ^43^ The flow chart for the study selection process is illustrated in *Figure* *1*.

### Study characteristics

The general characteristics of the 13 qualified articles are illustrated in *Table* *S5*. Selected eligible trials enrolled 1229 (619 intervention and 610 control) participants with ages ranging from 50.3 to 66.7 years. Except for one trial,^21^ which had a cross‐over design, all studies incorporated a parallel design. All studies were conducted on both females and males. Six trials used chemotherapy and radiotherapy as concurrent treatments,^18^, ^20^, ^22^, ^39^, ^41^, ^43^ and four studies used chemotherapy^20^, ^23^, ^25^, ^42^ as a concurrent treatment to MA. The duration of the studies ranged from 2 to 18 weeks. A range of supplementation doses were used between studies to determine the effect of MA, ranging from 160 to 800 mg/day. While a 320‐mg/day intervention was the most frequent dosage among trials. Moreover, two studies (Vadell et al. 1998 and Beller et al. 1997) reported two effect sizes that used low‐dose and high‐dose megestrol.^23^, ^43^

## Findings from meta‐analyses

### The effect of MA on body weight

In total, 12 trials,^18^, ^19^, ^20^, ^21^, ^22^, ^23^, ^24^, ^39^, ^40^, ^41^, ^42^, ^43^ including 14 effect sizes derived from 1,369 patients (intervention = 689, control = 680), provided data on the effect of MA on body weight. We did the highest versus lowest analysis and observed that body weight had not significantly increased following MA supplementation (MD: 0.64 kg, 95% CI [−0.11, 1.38], *P* = 0.093), with significant between‐study heterogeneity (*I* ^2^ = 69.1, *P* < 0.001) (*Table* *1* and *Figure* *S1*). To find the source of heterogeneity, we implemented subgroup analysis and discovered that duration ≤8 weeks (*I* ^2^ = 0.0%, *P* = 0.721) was the potential source of heterogeneity.

**Table 1:** The effect of megestrol acetate supplementation in cancer patients with anorexia–cachexia syndrome

	Pairwise meta‐analysis					Dose–response meta‐analysis					
	Studies, *n*	MD (95% CI)	*P value*	*I* ^2^, %	*P*_heterogeneity_	Dose, mg/day	Studies, *n*	MD (95% CI)	*P* value	*I* ^2^, %	*P*_heterogeneity_
Weight (kg)	14	0.64 [−0.11, 1.38]	0.093	69.1	<0.001	200	14	0.44 [0.13, 0.74]	0.005	97.1	<0.001
Appetite (score)	3	0.29 [−0.05, 0.64]	0.096	18.3	0.294	—	—	—	—	—	—
Fatigue (score)	2	0.14 [−0.09, 0.36]	0.236	0.0	0.379	—	—	—	—	—	—
EORTC QLQ‐C30 (score)	2	1.15 [0.76, 1.54]	<0.001	0.0	0.341	—	—	—	—	—	—

Abbreviations: CI, confidence interval; EORTC‐QLQ‐C30, European Organization for Research and Treatment of Cancer Quality of Life Questionnaire C30; MD, mean difference.

Findings from the subgroup analyses showed a significant increase in body weight following short term intervention (≤8 weeks) (MD: 0.62 kg, 95% CI [0.28, 0.96]; *P* < 0.001), and a combination of radio/chemotherapy as concurrent treatment (MD: 0.19 kg, 95% CI [−0.04, 0.35]; *P* = 0.015) (*Table* *2*).

**Table 2:** Result of subgroup analysis of included studies in meta‐analysis

Sub‐grouped by	No. of trials	Effect size^a^	95% CI, *P* value	*I* ^2^ (%)	*P* for heterogeneity	*P* for between subgroup heterogeneity
Weight (all trials)	14	0.64	[−0.11, 1.38], 0.093	69.1	<0.001	
Duration						**0.010**
≤8 weeks	3	0.62	[0.28, 0.96], <0.001	0.0	0.721	
>8 weeks	12	0.12	[−0.08, 0.31], 0.249	62.2	0.003	
Dose						**0.305**
Low dose (≤320 g/day)	8	0.17	[−0.06, 0.41], 0.142	55.0	0.030	
High dose (>320 g/day)	6	0.21	[−0.12, 0.54], 0.206	70.7	0.004	
Type of control group						**0.984**
Placebo	11	0.21	[−0.03, 0.45], 0.086	69.9	0.001	
Medication	3	0.19	[−0.02, 0.41], 0.082	0.0	0.818	
Concurrent treatment						**0.341**
Chemotherapy	3	0.07	[−0.41, 0.54], 0.780	72.2	0.027	
Radio (chemo)therapy	6	0.19	[0.04, 0.35], 0.015	5.2	0.383	
Not reported	5	0.23	[−0.29, 0.76], 0.387	79.0	0.001	

^a^ Calculated by random‐effects model.

*Note*: Findings from the subgroup analyses showed a significant increase in body weight following short‐term intervention (≤8 weeks) (shown in bold).

Abbreviations: CI, confidence interval.

Each trial was omitted step‐by‐step from the pooled analysis to distinguish the bearing of each individual study on the pooled effect size. After excluding the RCTs conducted by McMillan (1994)^40^ and Vadell (low dose) (1998),^23^ a change was observed in the pooled effect size (*Figure* *S2*). The evaluation of publication bias based on Egger's test and Begg's test revealed no evidence of publication bias (Egger's test = 0.864 and Begg's test = 0.959). Although Egger's and Begg's tests failed to show evidence of publication bias, according to our funnel plot, an asymmetric publication bias was seen (*Figure* *S3*). So that it can be said our results may be overestimated due to publication bias.

**Figure 2:**
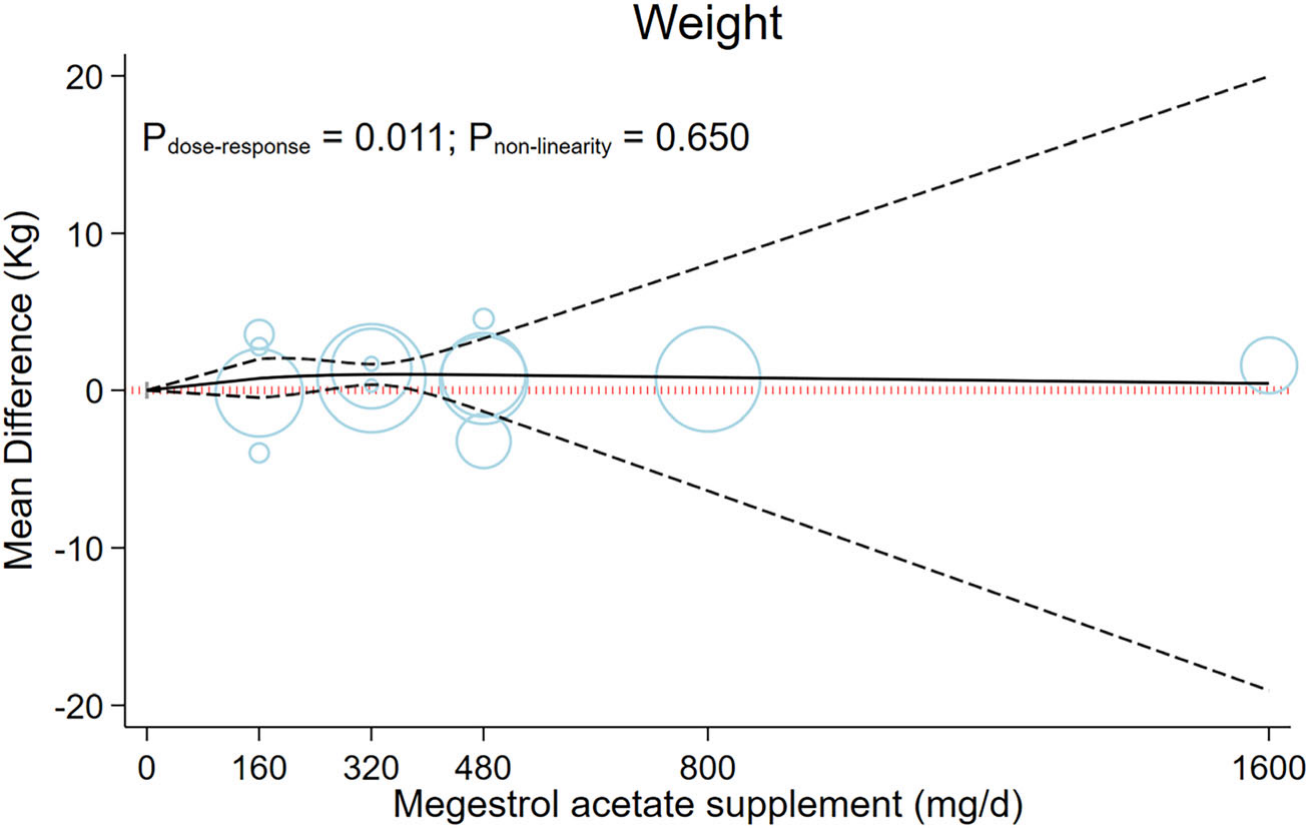
The effects of different doses of megestrol acetate supplementation on weight form the non‐linear dose response meta‐analysis.

A linear dose–response meta‐analysis indicated that each 200‐mg/day increment in MA consumption had a significant increase in weight gain (MD: 0.44; 95% CI [0.13, 0.74], *P* = 0.005; *I* ^2^ = 97.1, *P*_het_ < 0.001; *n* = 14 trials; *Figure* *S4*). Dose‐dependent effects of MA on weight gain are illustrated in *Figure* *2* and *Table* *1*. We discovered that there was a positive linear association between increased weight gain among patients treated with an increased dose of the MA supplement (*P*_*non‐linearity*_ = 0.650, *P*_*dose–response*_ = 0.011). However, the greatest effect on weight gain was observed in 320 mg of MA supplementation daily (MD_320mg/day_: 1.01, 95% CI [0.35, 1.67]; *Figure* *2* and *Table* *3*).

**Table 3:** The effects of different doses of megestrol acetate supplementation for cancer‐related anorexia/cachexia form the non‐linear dose–response meta‐analysis (mean difference and 95% confidence interval)

Megestrol acetate supplements (mg/day)	0 (Ref)	160	320	480	800	1,600
Weight (kg)	0	0.77 [−0.47, 2.00]	1.01 [0.35, 1.67]	0.98 [−1.34, 3.30]	0.83 [−6.37, 8.02]	0.44 [−19.09, 19.97]

We deemed very low certainty for the effect of MA on body weight. We downgraded the evidence certainty due to the risk of bias (unclear random sequence generation, allocation concealment and selective outcome reporting), imprecision (effect size did not surpass the MCID), publication bias (an asymmetric funnel plot) and inconsistency (72% heterogeneity) (*Table* *S6*).

### The effect of MA on appetite

A combination of 3 effect sizes for appetite score,^21^, ^41^, ^42^ which included 163 participants (intervention = 84, control = 79), showed that supplementation with MA had a non‐significant increase in appetite scores (MD: 0.29, 95% CI [−0.05, 0.64], *P* = 0.096) without any between‐study heterogeneity (*I* ^2^ = 18.3%, *P* = 0.294, *Figure* *S5*). The sensitivity analysis results showed that the overall effect was changed after excluding the Madeddu (2012) study^42^ (*Figure* *S6*).

Regarding appetite, very low certainty was found in the MA supplementation group due to the risk of bias (unclear blinding of participants or personnel, as well as blinding of the assessment outcome), and imprecision (both due to the insufficient sample size and the fact that the effect size did not exceed the MCID) (*Table* *S6*).

### The effect of MA on fatigue

Two eligible^24^, ^42^ trials, including 300 participants (150 intervention and 150 control), examined the effect of MA supplementation on fatigue. Combining their findings based on the random‐effects model, we found that fatigue scores had a non‐significant increase following MA supplementation in comparison to the control group (MD: 0.14, 95% CI [−0.09, 0.36], *P* = 0.236), without any significant heterogeneity between trials (*I* ^2^ = 0.0%, *P* = 0.379; *Figure* *S7*). We found no significant effects of any individual study on the pooled effect size.

We deemed very low certainty for the effect of MA on fatigue. We downgraded the evidence certainty due to the risk of bias (without blinding of participants or personnel, as well as blinding of the assessment outcome, incomplete outcome data, and other source of bias), and imprecision (both due to the insufficient sample size and the fact that the effect size did not exceed the MCID) (*Table* *S6*).

### The effect of MA on EORTC QLQ‐C30 (score)

The impact of MA supplementation on the EORTC QLQ‐C30 was assessed in two trials^25^, ^42^ comprising 176 participants (intervention = 87, control = 89). The pooled estimates demonstrated that supplementation with MA had a significant effect in EORTC QLQ‐C30 (MD: 1.15, 95% CI [0.76, 1.54], *P* < 0.001), with no evidence of significant between‐study heterogeneity (*I* ^2^ = 0.0%, *P* = 0.341; *Figure* *S8*). Besides, results from sensitivity analysis revealed no alteration in the overall effect size after omitting individual trials one by one.

Regarding the EORTC QLQ‐C30 score, very low certainty was found in the MA supplementation group due to the risk of bias (unclear blinding of participants or personnel, as well as blinding of the assessment outcome, incomplete outcome data and other source of bias), and imprecision (both due to the insufficient sample size and the fact that the effect size did not exceed the MCID) (*Table* *S6*).

### Risk of biases and grading the evidence

According to the Cochrane RoB tool for RCTs, only one study was regarded to have ‘some concerns,’ and the remaining 12 trials were considered to have ‘high risk of bias’ (*Table* *S3*).

For the general title of randomization to MA or control, an unclear risk for sequence generation was discovered in 54% of trials and also, 77% of trials were found to have an unclear risk because they did not clearly describe allocation concealment. Three articles^22^, ^23^, ^24^ were considered to have a low risk for blinding participants and personnel and blindings of outcome assessors, researchers or caregivers, whereas, in seven trials, no blindings were found either for participants and personnel or outcome assessment, leading to incrementing detection bias. Regarding incomplete outcome data, low risk was discovered in only 38.4% of the trials because they had no missing data and reported the selected outcomes. Under the selective reporting domain, unclear and low risks were found in nine and four trials, respectively. Regarding other biases, only one study had low risk,^40^ while the remaining had unclear risk because this domain was not described or high risk because of certain concerns such as funding (not reported), conflict of interest or no date on the validity of the outcome measure.

## Discussion

In the present systematic review and meta‐analysis, we combined available records from 13 RCTs, comprising 1229 (619 intervention and 610 control) participants from different countries (Italy, USA, Germany, Turkey, Canada, Spain, UK, Australia, Sweden and China), which investigated the effects of MA in cancer patients with anorexia–cachexia syndrome. Our results indicated that MA supplementation significantly increased EORTC QLQ‐C30 scores (GRADE: very low). However, this result was insignificant for body weight, fatigue and appetite. Although we found no significant effect on body weight and appetite in the main analysis, after the elimination of two trials by Mc Millane et al.^40^ and Vadell et al.^23^ for body weight and Madeddu et al.^42^ for appetite, change was observed in the previous results. Low number of participants, lack of blinding, participants in different stages of cancer, differences in the site of cancer involvement and different doses of megestrol used in the trials may result in major differences between them and comparative studies. Findings from the dose–response analysis showed that each 200 mg/day increment in MA consumption causes a significant increase in weight gain (GRADE: low). To the best of our knowledge, this is the first comprehensive dose–response meta‐analysis to evaluate the effect of MA supplementation in cancer patients with anorexia–cachexia syndrome.

Currently, MA is the only approved drug in Europe for the treatment of cancer cachexia, making it one of the most widely prescribed drugs in the field. A potential explanation for its anticachectic mechanism is the stimulation of appetite through direct and indirect routes, as well as modulating glucocorticoid activity, downregulating proinflammatory cytokines and increasing food intake via neuropeptide Y release.^44^ As synthetic progesterone, MA may increase body fat stores and stimulate appetite, but it does not increase lean body mass.^45^ Studies have noted that after MA was discontinued in participants, weight loss reverted to its previous state, suggesting that the metabolic effects of MA are likely to be mediated by its anti‐inflammatory properties.^46^ It is common for patients to experience headaches and nausea after taking progestins, as well as thrombosis at high doses.

Our findings relating to MA and appetite are in contrast with several previous systematic reviews and meta‐analyses. A meta‐analysis performed in 2013^15^ identified a significant improvement in appetite among patients treated with MA. In line with our findings, the results of this study were significant regarding weight gain, although there was significant heterogeneity in the results obtained from this review. Ruiz‐Garcia et al.^16^ conducted a meta‐analysis consisting of 38 trials and revealed that in trials where MA was compared with a placebo, the overall results showed that participants who were exposed to MA gained weight but did not improve their quality of life. Contrary to this, our analysis showed that the use of MA compared with a control did not have a significant effect on weight gain in patients with cancer‐related anorexia/cachexia. This discrepancy with the mentioned review (Ruiz‐Garcia et al.) may be a result of the review including patients with AIDS, degenerative diseases, anorexia nervosa and other terminal illnesses. In line with our findings, a recent systematic review and meta‐analysis^17^ also failed to find a significant effect of supplementation with MA on weight gain, but we revealed a significant effect on EORTC QLQ‐C30 scores. The analysis performed in this review was done by separating high‐dose (>320 mg/day) and low‐dose (320 mg/day) MA, which was not significant in any of the cases. Notably, we included several studies^18^, ^19^, ^20^, ^21^, ^22^, ^23^, ^24^, ^25^ that were missed in the earlier 2022 review. Compared with the previous meta‐analysis, our review is the first to investigate the effect of MA supplementation on fatigue,. Furthermore, we performed a dose–response analysis to clarify the effect of MA supplementation on weight gain and appetite scores.

Several strengths can be identified in the current review. First, this is the initial comprehensive meta‐analysis on the effects of MA on fatigue in cancer patients with anorexia–cachexia syndrome. Second, a dose–response analysis was conducted to evaluate the linear and non‐linear effects of MA on body weight. Thirdly, given that only RCTs were included in the review, we can get a good sense of a causal relationship from the findings. Moreover, sensitivity analyses revealed that no single study or group of studies affected the findings significantly. Notably, the GRADE system was adopted to evaluate the overall quality of the evidence, which generally demonstrated a moderate certainty of evidence based on the analysed trials. It should be noted that the analysis had a number of limitations. Some studies failed to report on the type of MA consumed, which can potentially affect its bioavailability and effectiveness. The limited number of studies included in the analysis of some of the reported variables (appetite, fatigue, *EORTC QLQ‐C30*). Based on our analysis for body weight, there was significant between‐study heterogeneity. Both the doses of MA and the duration of the interventions varied across the included studies. Our subgroup analysis showed that having a control group that consisted of patients who were exposed to a range of therapies (chemotherapy, radiotherapy/chemotherapy) and different durations of intervention (≤8 weeks, >8 weeks) were potential sources of heterogeneity. Moreover, Although Egger regression failed to show evidence of publication bias, according to our funnel plot, an asymmetric publication bias was seen so that our results may be overestimated due to publication bias. In addition, the majority of included studies were conducted in European countries, possibly limiting the generalizability of results.

## Conclusion

In conclusion, with very low certainty of evidence, MA supplementation might have a favourable effect on increasing quality of Life score in cancer patients with anorexia–cachexia syndrome. In contrast, with very low certainty, the effect of MA on body weight, appetite and fatigue was not significant according to the highest versus lowest analysis. Although liner dose–response analysis indicated a significant association between each 200 mg/day increment in MA supplementation with weight gain, the magnitude of the effect was small. The effects of MA consumption on patients with anorexia–cachexia syndrome will need to be confirmed in high‐quality clinical trials with larger sample sizes and adequate durations.

## Conflict of interest statement

The authors declare no conflict of interest.

## Funding

This research was financially supported by Haematology‐ Oncology and Stem Cell Transplantation Research Center of Tehran University of Medical Sciences (code: 62676 and IR.TUMS.HORCSCT.REC.1401.038).

## Supporting information


**Table S1.** Search strategies including the key terms and the queries for each database
**Table S2**: Reason for exclusion of retrieved articles
**Table S3.** Cochrane Risk of Bias Assessment
**Table S4**: Sensitivity analyses of the use of correlation coefficients of 0.25 and 0.75
**Table S5:** Characteristics of eligible studies on the effects of megestrol acetate supplementation in cancer patients with anorexia‐cachexia syndrome
**Table S6.** The GRADE evidence quality for each outcome.
**Figure S1.** Forest plot of the effect of megestrol acetate supplementation on weight using random effects model. MD: mean difference, CI: confidence interval.
**Figure S2.** Forest plots show sensitivity analysis results of weight.
**Figure S3.** Funnel plot for evaluation publication bias of weight.
**Figure S4.** Weighted mean difference of weight for a 200 mg/d increment in megestrol acetate supplementation using random effects model. MD: mean difference, CI: confidence interval.
**Figure S5.** Forest plot of the effect of megestrol acetate supplementation on appetite using random effects model. MD: mean difference, CI: confidence interval.
**Figure S6.** Forest plots show sensitivity analysis results of appetite.
**Figure S7.** Forest plot of the effect of megestrol acetate supplementation on fatigue using random effects model. MD: mean difference, CI: confidence interval.
**Figure S8.** Forest plot of the effect of megestrol acetate supplementation on European Organization for Research and Treatment of Cancer Quality of Life Questionnaire C30 using random effects model. MD: mean difference, CI: confidence interval.
