# Supplementary material for: Dose‐dependent effect of megestrol acetate supplementation in cancer patients with anorexia–cachexia syndrome: A meta‐analysis
Source: J Cachexia Sarcopenia Muscle. 2024 Jun 20;15(4):1254–63. doi: 10.1002/jcsm.13500 (PMC11294013; doi:10.1002/jcsm.13500)
Supplement: Supplementary file 1 — Table S1. Search strategies including the key terms and the queries for each database Table S2: Reason for exclusion of retrieved articles Table S3. Cochrane Risk of Bias Assessment Table S4: Sensitivity analyses of the use of correlation coefficients of 0.25 and 0.75 Table S5: Characteristics of eligible studies on the effects of megestrol acetate supplementation in cancer patients with anorexia‐cachexia syndrome Table S6. The GRADE evidence quality for each outcome. Figure S1. Forest plot of the effect of megestrol acetate supplementation on weight using random effects model. MD: mean difference, CI: confidence interval. Figure S2. Forest plots show sensitivity analysis results of weight. Figure S3. Funnel plot for evaluation publication bias of weight. Figure S4. Weighted mean difference of weight for a 200 mg/d increment in megestrol acetate supplementation using random effects model. MD: mean difference, CI: confidence interval. Figure S5. Forest plot of the effect of megestrol acetate supplementation on appetite using random effects model. MD: mean difference, CI: confidence interval. Figure S6. Forest plots show sensitivity analysis results of appetite. Figure S7. Forest plot of the effect of megestrol acetate supplementation on fatigue using random effects model. MD: mean difference, CI: confidence interval. Figure S8. Forest plot of the effect of megestrol acetate supplementation on European Organization for Research and Treatment of Cancer Quality of Life Questionnaire C30 using random effects model. MD: mean difference, CI: confidence interval. [file JCSM-15-1254-s001.docx]

**Dose-dependent effect of megestrol acetate supplementation in cancer patients with anorexia-cachexia syndrome: A systematic review and dose-response Meta-analysis of randomized controlled trials**

Online Supplementary Material

Supporting Materials including Supplementary Tables 1-6, Supplementary Figures 1-8

| **Table S1.** Search strategies including the key terms and the queries for each database | |
| --- | --- |
| **Database**  **6/22/2023** | **key terms and the queries** |
| PubMed  (n=376) | #1 ("Megestrol"[Title/Abstract] OR "Megestrol Acetate"[Title/Abstract] OR ("Megestrol"[MeSH Terms] OR "Megestrol Acetate"[MeSH Terms] OR "megestrol caproate"[Supplementary Concept]))  #2 ("Neoplasms"[MeSH Terms] OR "cancer"[Title/Abstract])  #3 "Randomized Controlled Trial"[Publication Type] OR "Randomized Controlled Trial"[Title/Abstract] OR "Controlled Clinical Trial"[Title/Abstract] OR "Controlled Clinical Trial"[Publication Type] OR "clinical trial"[Title/Abstract] OR "randomized"[Title/Abstract] OR "placebo"[Title/Abstract] OR "intervention studies"[Title/Abstract] OR "intervention"[Title/Abstract] OR "controlled trial"[Title/Abstract] OR "random"[Title/Abstract] OR "randomly"[Title/Abstract] OR "blind"[Title/Abstract] OR "RCT"[Title/Abstract] OR "trial"[Title/Abstract]  #4 #1 AND #2 AND #3 |
| Web of Science (ISI)  (n=1057) | #1 TOPIC: (" megestrol”) OR TOPIC: (" Megestrol Acetate ")  #2 TOPIC: (" Neoplasms") OR TOPIC: (" cancer")  #3 TOPIC: ("randomized controlled trial") OR TOPIC: ("clinical trial") OR TOPIC: ("randomized") OR TOPIC: ("placebo") OR TOPIC: ("intervention studies") OR TOPIC: ("intervention") OR TOPIC: ("controlled trial") OR TOPIC: ("random") OR TOPIC: ("randomly") OR TOPIC: ("blind") OR TOPIC: ("RCT") OR TOPIC: ("trial")  #4 #1 AND #2 AND #3 |
| Scopus  (n=2131) | #1 ( TITLE-ABS-KEY ( neoplasms ) OR TITLE-ABS-KEY ( cancer ) )  #2 ( TITLE-ABS-KEY ( megestrol ) OR TITLE-ABS-KEY ( "Megestrol Acetate" ) )  #3 TITLE-ABS-KEY ( "randomized controlled trial" ) OR TITLE-ABS-KEY ( "clinical trial" ) OR TITLE-ABS-KEY ( "randomized" ) OR TITLE-ABS-KEY ( "placebo" ) OR TITLE-ABS-KEY ( "intervention studies" ) OR TITLE-ABS-KEY ( "intervention" ) OR TITLE-ABS-KEY ( "controlled trial" ) OR TITLE-ABS-KEY ( "random" ) OR TITLE-ABS-KEY ( "randomly" ) OR TITLE-ABS-KEY ( "blind" ) OR TITLE-ABS-KEY ( "RCT" ) OR TITLE-ABS-KEY ( "trial " )  #4 #1 AND #2 AND #3 |
| ISRCTN registry  (n=12) | “megestrol acetate” AND “cancer” |
| ClinicalTrials.gov  (n=54) | “Neoplasms” OR “cancer” [DISEASE] AND "Megestrol OR Megestrol acetate" [TREATMENT] |
| ICTRP  (n=14) | “megestrol acetate” AND “cancer” |

| **Table S2**: Reason for exclusion of retrieved articles | |
| --- | --- |
| References | Reason for exclusion |
| 1. Loprinzi CL, Ellison NM, Schaid DJ, Krook JE, Athmann LM, Dose AM, et al. Controlled trial of megestrol acetate for the treatment of cancer anorexia and cachexia. Journal of the National Cancer Institute. 1990;82(13):1127-32. 2. Jatoi A, Rowland K, Loprinzi CL, et al. An Eicosapentaenoic Acid Supplement Versus Megestrol Acetate Versus Both for Patients With Cancer-Associated Wasting: A North Central Cancer Treatment Group and National Cancer Institute of Canada Collaborative Effort. Journal of Clinical Oncology. 2004;22(12):2469-2476. 3. Lai Y-L, Fang F-M, Yeh C-Y. Management of anorexic patients in radiotherapy: A prospective randomized comparison of megestrol and prednisolone. Journal of Pain and Symptom Management. 1994;9(4):265-268. 4. Rowland KM, Loprinzi CL, Shaw EG, et al. Randomized double-blind placebo-controlled trial of cisplatin and etoposide plus megestrol acetate/placebo in extensive-stage small-cell lung cancer: A North Central Cancer Treatment Group study. Journal of Clinical Oncology. 1996;14(1):135-141. 5. Ahlgren JD, Ellison NM, Gottlieb RJ, Laluna F, Lokich JJ, Sinclair PR, et al. Hormonal palliation of chemoresistant ovarian cancer: three consecutive phase II trials of the Mid-Atlantic Oncology Program. J Clin Oncol. 1993;11(10):1957-68. 6. Buzdar A, Douma J, Davidson N, Elledge R, Morgan M, Smith R, et al. Phase III, multicenter, double-blind, randomized study of letrozole, an aromatase inhibitor, for advanced breast cancer versus megestrol acetate. Journal of Clinical Oncology. 2001;19(14):3357-66. 7. Buzdar AU, Jones SE, Vogel CL, Wolter J, Plourde P, Webster A. A Phase III trial comparing anastrozole (1 and 10 milligrams), a potent and selective aromatase inhibitor, with megestrol acetate in postmenopausal women with advanced breast carcinoma. Cancer. 1997;79(4):730-9. 8. Currow DC, Glare P, Louw S, Martin P, Clark K, Fazekas B, et al. A randomised, double blind, placebo-controlled trial of megestrol acetate or dexamethasone in treating symptomatic anorexia in people with advanced cancer. Scientific Reports. 2021;11(1). 9. Cuvelier GD, Baker TJ, Peddie EF, Casey LM, Lambert PJ, Distefano DS, et al. A randomized, double-blind, placebo-controlled clinical trial of megestrol acetate as an appetite stimulant in children with weight loss due to cancer and/or cancer therapy. Pediatr Blood Cancer. 2014;61(4):672-9. 10. Dombernowsky P, Smith I, Falkson G, Leonard R, Panasci L, Bellmunt J, et al. Letrozole, a new oral aromatase inhibitor for advanced breast cancer: Double-blind randomized trial showing a dose effect and improved efficacy and tolerability compared with megestrol acetate. Journal of Clinical Oncology. 1998;16(2):453-61. 11. Goss PE, Winer EP, Tannock IF, Schwartz LH. Randomized phase III trial comparing the new potent and selective third- generation aromatase inhibitor vorozole with megestrol acetate in postmenopausal advanced breast cancer patients. Journal of Clinical Oncology. 1999;17(1):52-63. 12. Jatoi A, Windschitl HE, Loprinzi CL, Sloan JA, Dakhil SR, Mailliard JA, et al. Dronabinol versus megestrol acetate versus combination therapy for cancer-associated anorexia: A North Central Cancer Treatment Group study. Journal of Clinical Oncology. 2002;20(2):567-73. 13. Jonat W, Howell A, Blomqvist C, Eiermann W, Winblad G, Tyrrell C, et al. A randomised trial comparing two doses of the new selective aromatase inhibitor anastrozole (Arimidex)* with megestrol acetate in postmenopausal patients with advanced breast cancer. European Journal of Cancer. 1996;32(3):404-12. | Without sufficient data |
| 1. Dixon AR, Jackson L, Chan S, Haybittle J, Blamey RW. A randomised trial of second-line hormone vs single agent chemotherapy in tamoxifen resistant advanced breast cancer. Br J Cancer. 1992;66(2):402-4. 2. Bezwoda WR, Gudgeon A, Falkson G, Jordaan JP, Goedhals L. Fadrozole versus megestrol acetate: A double-blind randomised trial in advanced breast cancer. Oncology. 1998;55(5):416-20. 3. Fleming GF, Filiaci VL, Marzullo B, Zaino RJ, Davidson SA, Pearl M, et al. Temsirolimus with or without megestrol acetate and tamoxifen for endometrial cancer: A gynecologic oncology group study. Gynecologic Oncology. 2014;132(3):585-92. 4. Giacomin A, Sergio A, Vanin V, Tartaro P, Paccagnella D, Mazzucco M, et al. Megestrol and embryonic extracts in the treatment of advanced hepatocellular carcinoma: A prospective randomized trial in the pre-sorafenib era. Hepatology Research. 2010;40(2):153-60. 5. Gill PG, Gebski V, Snyder R, Burns I, Levi J, Byrne M, et al. Randomized comparison of the effects of tamoxifen, megestrol acetate, or tamoxifen plus megestrol acetate on treatment response and survival in patients with metastatic breast cancer. Annals of Oncology. 1993;4(9):741-4. 6. Hillner BE, Radice D. Cost-effectiveness analysis of exemestane compared with megestrol in patients with advanced breast carcinoma. Cancer. 2001;91(3):484-9. 7. Ingle JN, Ahmann DL, Green SJ, Edmonson JH, Creagan ET, Hahn RG, et al. Randomized clinical trial of megestrol acetate versus tamoxifen in paramenopausal or castrated women with advanced breast cancer. American Journal of Clinical Oncology: Cancer Clinical Trials. 1982;5(2):155-60. 8. Kaufmann M, Bajetta E, Dirix LY, Fein LE, Jones SE, Cervek J, et al. Exemestane improves survival compared with megoestrol acetate in postmenopausal patients with advanced breast cancer who have failed on tamoxifen: Results of a double-blind randomised phase III trial. European Journal of Cancer. 2000;36(SUPPL. 4):86-7. 9. Kim YH, Choi HY, Jin S, Noh YH, Kim MJ, Park HJ, et al. Tolerability and Pharmacokinetics of Two Formulations of Megestrol Acetate under Fed Conditions in Healthy Volunteers. Clinical Therapeutics. 2015;37(2):439-47. 10. Lansiaux A, Salingue S, Dewitte A, Clisant S, Penel N. Circulating thrombospondin 1 level as a surrogate marker in patients receiving cyclophosphamide-based metronomic chemotherapy. Investigational New Drugs. 2012;30(1):403-4. 11. Leitzel K, Teramoto Y, Konrad K, Chinchilli VM, Volas G, Grossberg H, et al. Elevated serum c-erbB-2 antigen levels and decreased response to hormone therapy of breast cancer. Journal of Clinical Oncology. 1995;13(5):1129-35. 12. Löfgren L, Wallberg B, Wilking N, Fornander T, Rutqvist LE, Carlström K, et al. Tamoxifen and megestrol acetate for postmenopausal breast cancer: Diverging effects on liver proteins, androgens, and glucocorticoids. Medical Oncology. 2004;21(4):309-18. 13. Markovic S, Suman VJ, Dalton RJ, Woods JE, Fitzgibbons Jr RJ, Wold LE, et al. Randomized, placebo-controlled, phase III surgical adjuvant clinical trial of megestrol acetate (megace) in selected patients with malignant melanoma. American Journal of Clinical Oncology: Cancer Clinical Trials. 2002;25(6):552-6. 14. Muss HB, Wells HB, Paschold EH, Black WR, Cooper MR, Capizzi RL, et al. Megestrol acetate versus tamoxifen in advanced breast cancer: 5-year analysis--a phase III trial of the Piedmont Oncology Association. Journal of clinical oncology : official journal of the American Society of Clinical Oncology. 1988;6(7):1098-106. 15. Patel SR, Kvols LK, Hahn RG, Windschitl H, Levitt R, Therneau T. A phase II randomized trial of megestrol acetate or dexamethasone in the treatment of hormonally refractory advanced carcinoma of the prostate. Cancer. 1990;66(4):655-8. 16. Pilepich MV, Buzydlowski JW, John MJ, Rubin P, McGowan DG, Marcial VA. Phase II trial of hormonal cytoreduction with megestrol and diethylstilbestrol in conjunction with radiotherapy for carcinoma of the prostate: Outcome results of RTOG 83-07. International Journal of Radiation Oncology, Biology, Physics. 1995;32(1):175-80. 17. Russell CA, Green SJ, O'Sullivan J, Hynes HE, Budd GT, Congdon JE, et al. Megestrol acetate and aminoglutethimide/hydrocortisone in sequence or in combination as second-line endocrine therapy of estrogen receptor- positive metastatic breast cancer: A southwest oncology group phase III trial. Journal of Clinical Oncology. 1997;15(7):2494-501. 18. Sharifzadeh F, Aminimoghaddam S, Kashanian M, Fazaeli M, Sheikhansari N. A comparison between the effects of metformin and megestrol on simple endometrial hyperplasia. Gynecological Endocrinology. 2017;33(2):152-5. 19. Thürlimann B, Castiglione M, Hsu-Schmitz SF, Cavalli F, Bonnefoi H, Fey MF, et al. Formestane versus megestrol acetate in postmenopausal breast cancer patients after failure of tamoxifen: A phase III prospective randomised cross over trial of second-line hormonal treatment (SAKK 20/90). European Journal of Cancer Part A. 1997;33(7):1017-24. 20. Zang J, Hou M, Gou HF, Qiu M, Wang J, Zhou XJ, et al. Antiemetic activity of megestrol acetate in patients receiving chemotherapy. Support Care Cancer. 2011;19(5):667-73. 21. Zhang EN, Qu WQ, Liu L, Wang P, Zhong ZK, Fu AQ, et al. Clinical observation of preventive effect of ondansetron with or without megestrol acetate on chemotherapy-induced vomiting in gastrointestinal cancer patients. Chinese Journal of Cancer Prevention and Treatment. 2009;16(17):1329-31. 22. Chen HC, Leung SW, Wang CJ, Sun LM, Fang FM, Hsu JH. Effect of megestrol acetate and prepulsid on nutritional improvement in patients with head and neck cancers undergoing radiotherapy. Radiotherapy and oncology. 1997 Apr 1;43(1):75-9. | No relevant outcome reported |
| 1. Ansfield FJ, Davis Jr HL, Ellerby RA, Amirez GR. A clinical trial of megestrol acetate in advanced breast cancer. Cancer. 1974 Apr;33(4):907-10. 2. Creagan ET, Ingle JN, Schutt AJ, Schaid DJ. A prospective, randomized controlled trial of megestrol acetate among high-risk patients with resected malignant melanoma. American journal of clinical oncology. 1989 Apr 1;12(2):152-5. 3. Heckmayr M, Gatzemeier U. Megestrol acetate in cachectic patients with advanced bronchogenic cancer. Oncology Research and Treatment. 1990;13(4):285-7. 4. Patel SR, Kvols LK, Hahn RG, Windschitl H, Levitt R, Therneau T. A phase II randomized trial of megestrol acetate or dexamethasone in the treatment of hormonally refractory advanced carcinoma of the prostate. Cancer. 1990 Aug 15;66(4):655-8. 5. Kornblith AB, Hollis DR, Zuckerman E, Lyss AP, Canellos GP, Cooper MR, Herndon 2nd JE, Phillips CA, Abrams J, Aisner J. Effect of megestrol acetate on quality of life in a dose-response trial in women with advanced breast cancer. The Cancer and Leukemia Group B. Journal of clinical oncology. 1993 Nov;11(11):2081-9. 6. Loprinzi CL, Bernath AM, Schaid DJ, Malliard JA, Athmann LM, Michalak JC, Tschetter LK, Hatfield AK, Morton RF. Phase III evaluation of 4 doses of megestrol acetate as therapy for patients with cancer anorexia and/or cachexia. Oncology. 1994;51(Suppl. 1):2-7. 7. Ulutin HC, Arpaci F, Pak Y. Megestrol acetate for cachexia and anorexia in advanced non-small cell lung cancer: a randomized study comparing two different doses. Tumori Journal. 2002 Jul;88(4):277-80. 8. Licchetta A, Correale P, Migali C, Remondo C, Francini E, Pascucci A, Magliocca A, Guarnieri A, Savelli V, Piccolomini A, Carli AF. Oral metronomic chemo-hormonal-therapy of metastatic breast cancer with cyclophosphamide and megestrol acetate. Journal of Chemotherapy. 2010 Jun 1;22(3):201-4. 9. Sancho Cuesta JF, Fernandez PM, Ines CA, et al. Megestrol Acetate and Weight Loss in Advanced Cancer. 1993:1141. 10. Mcmillan D, Simpson J, Preston T, et al. Effect of megestrol acetate on weight loss, body composition and blood screen of gastrointestinal cancer patients. Clinical Nutrition. 1994;13(2):85-89. 11. Loprinzi CL, Michalak JC, Schaid DJ, et al. Phase III evaluation of four doses of megestrol acetate as therapy for patients with cancer anorexia and/or cachexia. Journal of Clinical Oncology. 1993;11(4):762767. 12. Heckmayr M, Gatzemeier U. Treatment of Cancer Weight Loss in Patients with Advanced Lung Cancer. Oncology. 1992;49(2):32-34. 13. Gebbia V, Testa A, Gebbia N. Prospective randomised trial of two dose levels of megestrol acetate in the management of anorexia-cachexia syndrome in patients with metastatic cancer. British Journal of Cancer. 1996;73(12):1576-1580 14. Mcmillan DC, Wigmore SJ, Wigmore KCH, O’Gorman P, Wright CE, Mcardle CS. A prospective randomized study of megestrol acetate and ibuprofen in gastrointestinal cancer patients with weight loss. British Journal of Cancer. 1999;79(3/4):495-500. 15. Navari RM, Brenner MC. Treatment of cancer-related anorexia with olanzapine and megestrol acetate: a randomized trial. Supportive Care in Cancer. 2009;18:951-956. 16. Kouchaki B, Janbabai G, Alipour A, Ala S, Borhani S, Salehifar E. Randomized double-blind clinical trial of combined treatment with megestrol acetate plus celecoxib versus megestrol acetate alone in cachexiaanorexia syndrome induced by GI cancers. Supportive Care in Cancer. 2018;26(7):2479-2489. 17. Wen H-S, Li X, Cao Y-Z, et al. Clinical Studies on the Treatment of Cancer Cachexia with Megestrol Acetate plus Thalidomide. Chemotherapy. 2013;58(6):461-467. 18. Abrams J, Aisner J, Cirrincione C, Berry DA, Muss HB, Cooper MR, et al. Dose-response trial of megestrol acetate in advanced breast cancer: Cancer and leukemia group B phase III study 8741. Journal of Clinical Oncology. 1999;17(1):64-73. 19. Alexieva-Figusch J, Blankenstein MA, Hop WC, Klijn JG, Lamberts SW, de Jong FH, et al. Treatment of metastatic breast cancer patients with different dosages of megestrol acetate; dose relations, metabolic and endocrine effects. Eur J Cancer Clin Oncol. 1984;20(1):33-40. 20. Amoroso D, Boccardo F, Balestrero M, Miglietta L, Brema F, Cellerino R, et al. Megestrol acetate plus alpha 2a interferon as second line therapy for postmenopausal patients with advanced breast cancer: Results of a multicentric phase II trial. Breast Cancer Research and Treatment. 1995;33(3):265-8. 21. Belinson JL, McClure M, Badger G. Randomized trial of megestrol acetate vs. megestrol acetate/tamoxifen for the management of progressive or recurrent epithelial ovarian carcinoma. Gynecologic Oncology. 1987;28(2):151-5. 22. Bines J, Dienstmann R, Obadia RM, Branco LGP, Quintella DC, Castro TM, et al. Activity of megestrol acetate in postmenopausal women with advanced breast cancer after nonsteroidal aromatase inhibitor failure: A phase ii trial. Annals of Oncology. 2014;25(4):831-6. 23. Cobau CD, Declercq K, Neuberg D, Ingle JN, Tormey DC. A randomized trial of megestrol acetate with or without premarin in the treatment of potentially responsive metastatic breast cancer: A study of the Eastern Cooperative Oncology Group (E2185). Cancer. 1996;77(3):483-9. 24. Dawson NA, Conaway M, Halabi S, Winer EP, Small EJ, Lake D, et al. A randomized study comparing standard versus moderately high dose megestrol acetate for patients with advanced prostate carcinoma: Cancer and leukemia group B study 9181. Cancer. 2000;88(4):825-34. 25. Eftekhar Z, Izadi-Mood N, Yarandi F, Shojaei H, Rezaei Z, Mohagheghi S. Efficacy of megestrol acetate (megace) in the treatment of patients with early endometrial adenocarcinoma: Our experiences with 21 patients. International Journal of Gynecological Cancer. 2009;19(2):249-52. 26. Fiorica JV, Brunetto VL, Hanjani P, Lentz SS, Mannel R, Andersen W. Phase II trial of alternating courses of megestrol acetate and tamoxifen in advanced endometrial carcinoma: A Gynecologic Oncology Group study. Gynecologic Oncology. 2004;92(1):10-4. 27. Goodwin JW, Green SJ, Giarritta S, Giguere JK, Hoelzer K, Bearden J, et al. Double blind phase III trial of placebo (P) vs. megestrol acetate (MA) 20 mg vs. MA 40 mg as treatment for symptoms of ovarian failure in breast cancer survivors: Initial results of Southwest Oncology Group S9626. Breast Cancer Research and Treatment. 2001;69(3):292. 28. Greig CA, Johns N, Gray C, MacDonald A, Stephens NA, Skipworth RJ, et al. Phase I/II trial of formoterol fumarate combined with megestrol acetate in cachectic patients with advanced malignancy. Support Care Cancer. 2014;22(5):1269-75. 29. Grunberg SM, Weiss MH. Lack of efficacy of megestrol acetate in the treatment of unresectable meningioma. Journal of Neuro-Oncology. 1990;8(1):61-5. 30. Heckmayr M, Gatzemeier U. Megestrol acetate in cachectic patients with advanced bronchogenic cancer. Onkologie. 1990;13(4):285-7. 31. Khuri FR, Fossella FV, Lee JS, Murphy WK, Shin DM, Markowitz AB, et al. Phase II trial of recombinant IFN-α2a with etoposide/cisplatin induction and interferon/megestrol acetate maintenance in extensive small cell lung cancer. Journal of Interferon and Cytokine Research. 1998;18(4):241-5. 32. Kornblith AB, Hollis DR, Zuckerman E, Lyss AP, Canellos GP, Cooper MR, et al. Effect of megestrol acetate on quality of life in a dose-response trial in women with advanced breast cancer. Journal of Clinical Oncology. 1993;11(11):2081-9. 33. Lambert CP, Sullivan DH, Freeling SA, Lindquist DM, Evans WJ. Effects of testosterone replacement and/or resistance exercise on the composition of megestrol acetate stimulated weight gain in elderly men: A randomized controlled trial. Journal of Clinical Endocrinology and Metabolism. 2002;87(5):2100-6. 34. Levitan N, Dowlati A, Craffey M, Tahsildar H, MacKay W, McKenney J, et al. A brief intensive cisplatin-based outpatient chemotherapy regimen with filgrastim and megestrol acetate support for advanced non-small cell lung cancer: Results of a phase II trial. Lung Cancer. 1998;22(3):227-34. 35. Licchetta A, Correale P, Migali C, Remondo C, Francini E, Pascucci A, et al. Oral metronomic chemo-hormonal-therapy of metastatic breast cancer with cyclophosphamide and megestrol acetate. Journal of Chemotherapy. 2010;22(3):201-4. 36. MacCiò A, Madeddu C, Gramignano G, Mulas C, Floris C, Sanna E, et al. A randomized phase III clinical trial of a combined treatment for cachexia in patients with gynecological cancers: Evaluating the impact on metabolic and inflammatory profiles and quality of life. Gynecologic Oncology. 2012;124(3):417-25. 37. Markman M, Kennedy A, Webster K, Kulp B, Peterson G, Belinson J. Phase I trial of paclitaxel plus megestrol acetate in patients with paclitaxel-refractory ovarian cancer. Clinical Cancer Research. 2000;6(11):4201-4. 38. Matin K, Egorin MJ, Ballesteros MF, Smith DC, Lembersky B, Day RS, et al. Phase I and pharmacokinetic study of vinblastine and high-dose megestrol acetate. Cancer Chemotherapy and Pharmacology. 2002;50(3):179-85. 39. McMillan DC, O'Gorman P, Fearon KCH, McArdle CS. A pilot study of megestrol acetate and ibuprofen in the treatment of cachexia in gastrointestinal cancer patients. British Journal of Cancer. 1997;76(6):788-90. 40. Muss HB, Case LD, Capizzi RL, Cooper MR, Cruz J, Jackson D, et al. High- versus standard-dose megestrol acetate in women with advanced breast cancer: a phase III trial of the Piedmont Oncology Association. J Clin Oncol. 1990;8(11):1797-805. 41. Neri B, Gemelli MT, Tarantini P, Benvenuti F, Khader A, Ludovici M, et al. The role of megestrol acetate in neoplastic anorexia and cachexia. Current Therapeutic Research. 1995;56(2):183-9. 42. Pandya KJ, Yeap BY, Weiner LM, Krook JE, Erban JK, Schinella RA, et al. Megestrol and tamoxifen in patients with advanced endometrial cancer: An Eastern Cooperative Oncology Group Study (E4882). American Journal of Clinical Oncology: Cancer Clinical Trials. 2001;24(1):43-6. 43. Parnes HL, Abrams JS, Simon Tchekmedyian N, Tait N, Aisner J. A phase I/II study of high-dose megestrol acetate in the treatment of metastatic breast cancer. Breast Cancer Research and Treatment. 1991;18(3):171-7. 44. Pawlicki M, Koralewski P. The role of megestrol acetate in the treatment of cachexia in cancer patients. Nowotwory. 1995;45(1):28-34. 45. Sikic BI, Scudder SA, Ballon SC, Soriero OM, Christman JE, Suey L, et al. High-dose megestrol acetate therapy of ovarian carcinoma: A phase II study by the Northern California Oncology Group. Seminars in Oncology. 1986;13(4 SUPPL. 4):26-32. 46. Skarlos DV, Fountzilas G, Pavlidis N, Beer M, Makrantonakis P, Aravantinos G, et al. Megestrol acetate in cancer patients with anorexia and weight loss a hellenic co-operative oncology group (heCOG) study. Acta Oncologica. 1993;32(1):37-41. 47. Tchekmedyian NS, Tait N, Abrams J, Aisner J. High-dose megestrol acetate in the treatment of advanced breast cancer. Seminars in Oncology. 1988;15(2 SUPPL. 1):44-9. 48. Ulutin HC, Arpaci F, Pak Y. Megestrol acetate for cachexia and anorexia in advanced non-small cell lung cancer: A randomized study comparing two different doses. Tumori. 2002;88(4):277-80. 49. Wang L. Abdominal acupuncture combined with megestrol acetate for advanced cancer-related anorexia: A randomized controlled trial (vol 29, pg 200, 2019). World Journal of Acupuncture-Moxibustion. 2021;31(3):248-. 50. Wang L, Wang YY. Abdominal acupuncture combined with megestrol acetate for advanced cancer-related anorexia: A randomized controlled trial. World Journal of Acupuncture-Moxibustion. 2019;29(3):200-5. 51. Yang B, Yierfulati G, Guan J, Chen X. EFFECT OF MEGESTROL ACETATE PLUS METFORMIN AS FERTILITY-SPARING TREATMENT FOR PATIENTS WITH ATYPICAL ENDOMETRIAL HYPERPLASIA AND WELL-DIFFERENTIATED ENDOMETRIAL CANCER. A RANDOMIZED CONTROLLED TRIAL. International Journal of Gynecological Cancer. 2019;29:A4-A5. 52. Yang BY, Gulinazi Y, Du Y, Ning CC, Cheng YL, Shan WW, et al. Metformin plus megestrol acetate compared with megestrol acetate alone as fertility-sparing treatment in patients with atypical endometrial hyperplasia and well-differentiated endometrial cancer: a randomised controlled trial. BJOG: An International Journal of Obstetrics and Gynaecology. 2020;127(7):848-57. 53. Tehranian A, Ghahghaei-Nezamabadi A, Arab M, Khalagi K, Aghajani R, Sadeghi S. The impact of adjunctive metformin to progesterone for the treatment of non-atypical endometrial hyperplasia in a randomized fashion, a placebo-controlled, double blind clinical trial. J Gynecol Obstet Hum Reprod. 2021;50(6):101863. | No control group |
| 1. Sikic BI, Scudder SA, Ballon SC, Soriero OM, Christman JE, Suey L, Ehsan MN, Brandt AE, Evans TL. High-dose megestrol acetate therapy of ovarian carcinoma: a phase II study by the Northern California Oncology Group. InSeminars in oncology 1986 Dec 1 (Vol. 13, No. 4 Suppl 4, pp. 26-32). | Seminars in Oncology |
| 1. Lundholm K, Gelin J, Hyltander A, Lonnroth C, Sandstrom R, Svaninger G. Anti-inflammatory Treatment May Prolong Survival in Undernourished Patients with Metastatic Solid Tumors. Cancer Research. 1994;54:5602-5606. 2. Bruera E, Strasser F, Palmer JL, et al. Effect of Fish Oil on Appetite and Other Symptoms in Patients With Advanced Cancer and Anorexia/Cachexia: A Double-Blind, Placebo-Controlled Study. Journal of Clinical Oncology. 2003;21(1):129-134. 3. Fearon KC, Barber MD, Moses AG, et al. Double-Blind, Placebo-Controlled, Randomized Study of Eicosapentaenoic Acid Diester in Patients With Cancer Cachexia. Journal of Clinical Oncology. 2006;24(21):3401-3407. 4. Temel JS, Abernethy AP, Currow DC, et al. Anamorelin in patients with non-small-cell lung cancer and cachexia (ROMANA 1 and ROMANA 2): results from two randomised, double-blind, phase 3 trials. Lancet Oncol 2016. 5. Temel JS, Abernethy AP, Currow DC, et al. Anamorelin in patients with non-small-cell lung cancer and cachexia (ROMANA 1 and ROMANA 2): results from two randomised, double-blind, phase 3 trials. The Lancet Oncology. 2016;17(4):519-531. 6. Hiura Y, Takiguchi S, Yamamoto K, et al. Effects of ghrelin administration during chemotherapy with advanced esophageal cancer patients. Cancer. 2012;118(19):4785-4794. 7. Takayama K, Katakami N, Yokoyama T, et al. Anamorelin (ONO-7643) in Japanese patients with nonsmall cell lung cancer and cachexia: results of a randomized phase 2 trial. Supportive Care in Cancer. 2016;24(8):3495-3505. 8. Garcia JM, Boccia RV, Graham CD, et al. Anamorelin for patients with cancer cachexia: an integrated analysis of two phase 2, randomised, placebo-controlled, double-blind trials. The Lancet Oncology. 2015;16(1):108-116. 9. Adachi S, Takiguchi S, Okada K, et al. Effects of Ghrelin Administration After Total Gastrectomy: A Prospective, Randomized, Placebo-Controlled Phase II Study. Gastroenterology. 2010;138(4):1312-1320. 10. Garcia JM, Friend J, Allen S. Therapeutic potential of anamorelin, a novel, oral ghrelin mimetic, in patients with cancer-related cachexia: a multicenter, randomized, double-blind, crossover, pilot study. Supportive Care in Cancer. 2013;21(1):129-137. 11. Dobs AS, Boccia RV, Croot CC, et al. Effects of enobosarm on muscle wasting and physical function in patients with cancer: a double-blind, randomised controlled phase 2 trial. The Lancet Oncology. 2013;14(4):335-345. 12. Gordon JN, Trebble TM, Ellis RD, Duncan HD, Johns T, Goggin PM. Thalidomide in the treatment of cancer cachexia: a randomised placebo controlled trial. Gut. 2005;54(4):540-545. 13. Simons JP, Aaronson NK, Vansteenkiste JF, et al. Effects of medroxyprogesterone acetate on appetite, weight, and quality of life in advanced-stage non-hormone-sensitive cancer: a placebo-controlled multicenter study. Journal of Clinical Oncology. 1996;14(4):1077-1084. 14. Strasser F, Luftner D, Possinger K, et al. Comparison of Orally Administered Cannabis Extract and Delta- 9-Tetrahydrocannabinol in Treating Patients With Cancer-Related Anorexia-Cachexia Syndrome: A Multicenter, Phase III, Randomized, Double-Blind, Placebo-Controlled Clinical Trial From the Cannabis- In-Cachexia-Study-Group. Journal of Clinical Oncology. 2006;24(21):3394-3400. 15. Wilkes E, Selby A, Cole A, Freeman J, Rennie M, Khan Z. Poor tolerability of thalidomide in end-stage oesophageal cancer. European Journal of Cancer Care. 2011;20:593-600. 16. Cerchietti LC, Navigante AH, Castro MA. Effects of Eicosapentaenoic and Docosahexaenoic n-3 Fatty Acids From Fish Oil and Preferential Cox-2 Inhibition on Systemic Syndromes in Patients With Advanced Lung Cancer. Nutrition and Cancer. 2007;59(1):14-20. 17. Lai V, George J, Richey L, et al. Results of a pilot study of the effects of celecoxib on cancer cachexia in patients with cancer of the head, neck, and gastrointestinal tract. Head & Neck. January 2008:67-74. 18. Katakami N, Uchino J, Yokoyama T, et al. Anamorelin (ONO-7643) for the treatment of patients with non-small cell lung cancer and cachexia: Results from a randomized, double-blind, placebo-controlled, multicenter study of Japanese patients (ONO-7643-04). Cancer. 2018;124(3):606-616. 19. Del Fabbro E, Dev R, Hui D, Palmer L, Bruera E. Effects of Melatonin on Appetite and Other Symptoms in Patients With Advanced Cancer and Cachexia: A Double-Blind Placebo-Controlled Trial. Journal of Clinical Oncology. 2013;31(10):1271-1276. 20. Neri B, Garosi VL, Intini C. Effect of medroxyprogesterone acetate on the quality of life of the oncologic patient. Anti-Cancer Drugs. 1997;8(5):459-465. 21. Beijer S, Hupperets PS, Borne BEVD, et al. Effect of adenosine 5¬-triphosphate infusions on the nutritional status and survival of preterminal cancer patients. Anti-Cancer Drugs. 2009;20(7):625-633. 22. Agteresch HJ, Dagnelie PC, van der Gaast A, Stijnen T, Wilson JHP. Randomized Clinical Trial of Adenosine 5-Triphosphate in Patients with Advanced Non-Small-Cell Lung Cancer. Journal of the National Cancer Institute. 2000;92(4):321-328. 23. Kornek G, Schenk T, Ludwig H, Hejna M, Scheithauer W. Placebo-Controlled Trial of Medroxy – progesterone Acetate in Gastrointestinal Malignancies and Cachexia. Onkologie. 1996;19:164-168. 24. Wright TJ, Dillon EL, Durham WJ, et al. A randomized trial of adjunct testosterone for cancer-related muscle loss in men and women. Journal of Cachexia, Sarcopenia and Muscle. 2018:1-15. 25. Xu Z, Yang B, Guan J, Shan W, Liao J, Shao W, Chen X. Comparison of the effect of oral megestrol acetate with or without levonorgestrel-intrauterine system on fertility-preserving treatment in patients with early-stage endometrial cancer: a prospective, open-label, randomized controlled phase II trial (ClinicalTrials. gov NCT03241914). Journal of Gynecologic Oncology. 2023 Jan;34(1). | No megestrol group |
| 1. Jing X, Chen J, Zhong G. Effects of navelbine and cisplatin combined with megestrol acetate on advanced non-small cell lung cancer. Chinese Journal of Clinical Oncology. 2008;35(4):189-92. | Chinese language |
| 1. Mantovani G, Macciò A, Madeddu C, Gramignano G, Serpe R, Massa E, Dessì M, Tanca FM, Sanna E, Deiana L, Panzone F. Randomized phase III clinical trial of five different arms of treatment for patients with cancer cachexia: interim results. Nutrition. 2008 Apr 1;24(4):305-13. 2. Mantovani G, Macciò A, Madeddu C, Serpe R, Massa E, Dessì M, Panzone F, Contu P. Randomized phase III clinical trial of five different arms of treatment in 332 patients with cancer cachexia. The Oncologist. 2010 Feb;15(2):200-11. | The intervention group received medroxy progesterone acetate |

**Table S3.** Cochrane Risk of Bias Assessment

| **Study** | **Random Sequence Generation** | **Allocation concealment** | **Blinding of participants and personnel** | **Blinding of outcome assessment** | **Incomplete outcome data** | **Selective outcome reporting** | **Other sources of bias** | **Overall quality** |
| --- | --- | --- | --- | --- | --- | --- | --- | --- |
| Madeddu et al. (Italy; 2012) | L | U | H | H | L | U | H | High risk of bias |
| Loprinzi et al. (US; 1999) | U | U | H | H | H | U | U | High risk of bias |
| Fietkau et al. (Germany; 1997) | U | U | U | U | L | U | H | High risk of bias |
| De Conno et al. (Italy; 1998) | U | U | U | U | L | U | H | High risk of bias |
| Kanat et al. (Turkey; 2013) | U | U | H | H | U | U | H | High risk of bias |
| Bruera et al. (Canada; 1990) | L | U | U | U | H | L | U | High risk of bias |
| McQuellon et al. (NJ; 2002) | L | U | L | L | L | L | U | Some concerns |
| Vadell et al. (Spain; 1998) | U | U | L | L | U | U | U | High risk of bias |
| McMillan et al. (UK; 1994) | U | U | U | U | H | U | L | High risk of bias |
| Tchekmedyian et al. (US; 1991) | L | L | U | U | U | U | H | High risk of bias |
| Beller et al. (Australia; 1997) | U | L | U | U | L | U | U | High risk of bias |
| Westman et al. (Sweden; 1999) | L | L | L | L | H | L | U | High risk of bias |
| Ma et al.  (China, 2022) | L | U | U | U | H | L | H | High risk of bias |

**Abbreviations:** L, low risk of bias; H, high risk of bias; U, unclear risk of bias.

| **Table** **S4**: Sensitivity analyses of the use of correlation coefficients of 0.25 and 0.75 | | | |
| --- | --- | --- | --- |
| Outcome | MD (95% CI), P-value, I^2^, P-value _heterogeneity_ | | |
|  | Correlation coefficient used in the primary analysis | Correlation coefficient used in sensitivity analyses | |
|  | 0.5 | 0.25 | 0.75 |
| Weight (kg) | 0.64 (-0.11, 1.38), P=0.093, I^2^=69.1, P_het_<0.001 | 0.62 (-0.11, 1.35), P=0.096, I^2^=67.0, P_het_<0.001 | 0.68 (-0.10, 1.46), P=0.086, I^2^=74.0, P_het_<0.001 |
| Appetite (score) | 0.29 (-0.05, 0.64), P=0.086, I^2^=18.3, P_het_=0.294 | 0.29 (-0.05, 0.64), P=0.086, I^2^=18.3, P_het_=0.294 | 0.29 (-0.05, 0.64), P=0.086, I^2^=18.3, P_het_=0.294 |
| Fatigue (score) | 0.14 (-0.09, 0.36), P=0.236, I^2^=0.0, P_het_=0.379 | 0.11 (-0.11, 0.34), P=0.333, I^2^=0.0, P_het_=0.469 | 0.16 (-0.18, 0.48), P=0.334, I^2^=32.4, P_het_=0.224 |
| EORTC QLQ-C30 (score) | 1.15 (0.76, 1.54), P<0.001, I^2^=0.0, P_het_=0.341 | 1.15 (0.67, 1.62), P<0.001, I^2^=0.0, P_het_=0.437 | 2.08 (-1.33, 5.48), P=0.232, I^2^=44.8, P_het_=0.178 |

**Abbreviations:** MD; Mean Difference, CI; Confidence Interval, EORTC-QLQ-C30; European Organization for Research and Treatment of Cancer Quality of Life Questionnaire C30; IL-6, interleukin-6; TNF-alfa, tumor necrosis factor-alfa.

| **Table S5:** Characteristics of eligible studies on the effects of megestrol acetate supplementation in cancer patients with anorexia-cachexia syndrome | | | | | | | | | | | |
| --- | --- | --- | --- | --- | --- | --- | --- | --- | --- | --- | --- |
| **First author**  **(Country; Year)** | **RCT design**  **(blinding)** | **Underlying malignancy** | **Sex** | **Mean Age**  **(year)** | **Concurrent treatment** | **Sample size**  **(Megestrol acetate /Placebo)** | **Duration**  **(weeks)** | **Intervention** | | **Type of megestrol acetate** | **Outcomes** |
|  |  |  |  |  |  |  |  | **Treatment group** | **Control group** |  |  |
| Madeddu et al.  (Italy; 2012) | Parallel | Advanced stage tumor at any site | Both | 64.3 | Chemotherapy, hormone therapy (palliative or supportive care only) | 56  (27/29) | 16 | 320 mg/d | Placebo  Proinflam (Celecoxib 300 mg + L-carnitine 4 mg ) | NA | Weight, Fatigue, Appetite, quality of life |
| Loprinzi et al.  (US; 1999) | Parallel  (Double) | Advanced incurable cancer | Both | 66.7 | Chemotherapy (Cisplatin regimen, Noncisplatin regimen), radiation therapy | 228  (114/114) | 9 | 800 mg/d | Placebo  (Fluoxymesterone 20 mg) | Tablets | Appetite, Weight |
| Fietkau et al.  (Germany; 1997) | Parallel  (Double) | Carcinomas of the head and neck | Both | 50.3 | Chemotherapy, radiation therapy | 61  (31/30) | 18 | 160 mg/d | Placebo  (NR) | NA | Weight, Quality of life |
| De Conno et al.  (Italy; 1998) | Parallel  (Double) | Advanced non-hormone related tumour | Both | 61 | NA | 33  (17/16) | 2 | 320 mg/d | Placebo  (NR) | NA | Weight, Appetite, Quality of life |
| Kanat et al.  (Turkey; 2013) | Parallel | Advanced stage malignancy at any site | Both | 60.7 | Chemotherapy, hormone therapy or radiation  therapy with curative or palliative intent | 39  (21/18) | 12 | 320 mg/d | Placebo  (Meloxicam 15 mg, Eicosapentaenoic acid 2.2 g) | NA | Weight, Quality  of life |
| Bruera et al.  (Canada; 1990) | cross-over  (Double) | Advanced non-hormone-responsive tumors | Both | 62 | NA | 40  (20/20) | 2 | 480 mg/d | Placebo  (NR) | NA | Weight, Appetite |
| McQuellon et al.  (NJ; 2002) | Parallel  (Double) | Head, neck and lung cancer | Both | 62.9 | Radiotherapy, chemotherapy | 56  (28/28) | 12 | 160 mg/d | Placebo  (NR) | Tablets | Weight, Quality of life, Appetite |
| Vadell et al.  (Spain; 1998) | Parallel  (Double) | Advanced-stage cancer | Both | 64.1 | Chemotherapy | 101  (50/51) | 12 | 160 mg/d, 480 mg/d | Placebo  (NR) | Tablets | Weight, Quality-of life |
| McMillan et al.  (UK; 1994) | Parallel  (Double) | Metastatic gastrointestinal cancer | Both | 60 | NA | 26  (12/14) | 12 | 480 mg/d | Placebo  (NR) | NA | Weight |
| Tchekmedyian et al.  (US; 1991) | Parallel  (double) | Hormone-insensitive malignant lesions | Both | 63.4 | Chemotherapy, radiation therapy | 66  (36/30) | 4 | 1600 mg/d | Placebo  (NR) | Tablets | Weight, Appetite |
| Beller et al.  (Australia; 1997) | Parallel  (Double) | Advanced endocrine-insensitive cancer | Both | NR | Chemotherapy, radiation therapy | 159  (80/79) | 12 | 160 mg/d, 480 mg/d | Placebo  (NR) | Tablets | Weight, Quality of life |
| Westman et al.  (Sweden; 1999) | Parallel  (Double) | Advanced, Progressive, Hormone-insensitive Cancer | Both | NR | NA | 244  (123/121) | 12 | 320 mg/d | Placebo  (NR) | Tablets | Weight, Quality of life |
| Ma et al.  (China, 2022) | Parallel | Advanced tumors | Both | 56 | Chemotherapy | 120  (60/60) | 2 | 160 mg/d | 5-HT3 receptor antagonist plus dexamethasone | Tablets | Quality of life |

**Abbreviations:** NA, not applicant; NR, not report.

| **Table S6.** The GRADE evidence quality for each outcome. | | | | | | | | | | | |
| --- | --- | --- | --- | --- | --- | --- | --- | --- | --- | --- | --- |
| **Certainty assessment** | | | | | | | **No of patients** | | **Effect** | **Certainty** | **Importance** |
| **No of studies** | **Design** | **Risk of bias** | **Inconsistency** | **Indirectness** | **Imprecision** | **Other**  **considerations** | **Treatment group** | **Control group** | **MD (95%CI)** |  |  |
| **Weight (follow-up: range 2 weeks to 18 weeks)** | | | | | | | | | | | |
| 12 | randomised trials | very serious^a^ | serious^b^ | not serious | serious^c^ | dose-response gradient and publication bias strongly suspected^d^ | 689 | 680 | MD **0.44 kg higher** (0.13 higher to 0.74 higher) | ⨁◯◯◯ Very low | IMPORTANT |
| **Appetite (score) (follow-up: range 2 weeks to 16 weeks)** | | | | | | | | | | | |
| 3 | randomised trials | very serious^e^ | not serious^f^ | not serious | Serious^g^ | none | 84 | 79 | SMD **0.29 SD higher** (0.05 lower to 0.64 higher) | ⨁◯◯◯ Very low | IMPORTANT |
| **Fatigue (score) (follow-up: range 12 weeks to 16 weeks)** | | | | | | | | | | | |
| 2 | randomised trials | very serious^h^ | not serious^i^ | not serious | Serious^j^ | none | 150 | 150 | MD **0.14 higher** (0.09 lower to 0.36 higher) | ⨁◯◯◯ Very low | IMPORTANT |
| **EORTC QLQ-C30 (score) (follow-up: range 10 weeks to 16 weeks)** | | | | | | | | | | | |
| 2 | randomised trials | very serious^h^ | not serious^k^ | not serious | Serious^l^ | none | 87 | 89 | MD **1.15 higher** (0.76 higher to 1.54 higher) | ⨁◯◯◯ Very low | IMPORTANT |

**Abbreviations:** CI: confidence interval; MD: mean difference; SMD: standardized mean difference

#### Explanations

a. Very serious risk of bias due to the fact that most of the trials had unclear random sequence generation (n=7), allocation concealment (n=9) and selective outcome reporting (n=9). Downgraded.

b. Serious inconsistency since I2 = 97.1%, Phet < 0.001. Downgraded.

c. Optimal information size met (n=1369). The effect size (MD: 0.44) is near 1 kg and did not surpass the minimal clinically important difference for weight (MCID for adults: 2.5 kg). Downgraded.

d. According to our funnel plot, an asymmetric publication bias was seen. Downgraded for publication bias.

e. Very serious risk of bias due to the fact that all trials had unclear (n=2) or high (n=1) blinding of participants or personnel, as well as blinding of the assessment outcome. Downgraded.

f. Not serious inconsistency since I2 = 18.3%, Phet = 0.294. Not downgraded.

g. Optimal information size did not meet (n=163). The effect size (SMD: 0.29) did not surpass the minimal clinically important difference for appetite (MCID: 1.6). Downgraded.

h. Very serious risk of bias since two studies had high risk of bias. Downgraded.

i. Not serious inconsistency since I2 = 0%, Phet = 0.379 Not downgraded.

j. Optimal information size did not meet (n=300). The effect size (MD: 0.14) did not surpass the minimal clinically important difference for fatigue improvement (MCID: 0.9). Downgraded.

k. Not serious inconsistency since I2 = 0%, Phet = 0.341. Not downgraded.

l. Optimal information size did not meet (n=176). The effect size (MD: 1.15) did not surpass the minimal clinically important difference since patients should report 3-point increase on QLQ C-30 domains. Downgraded.


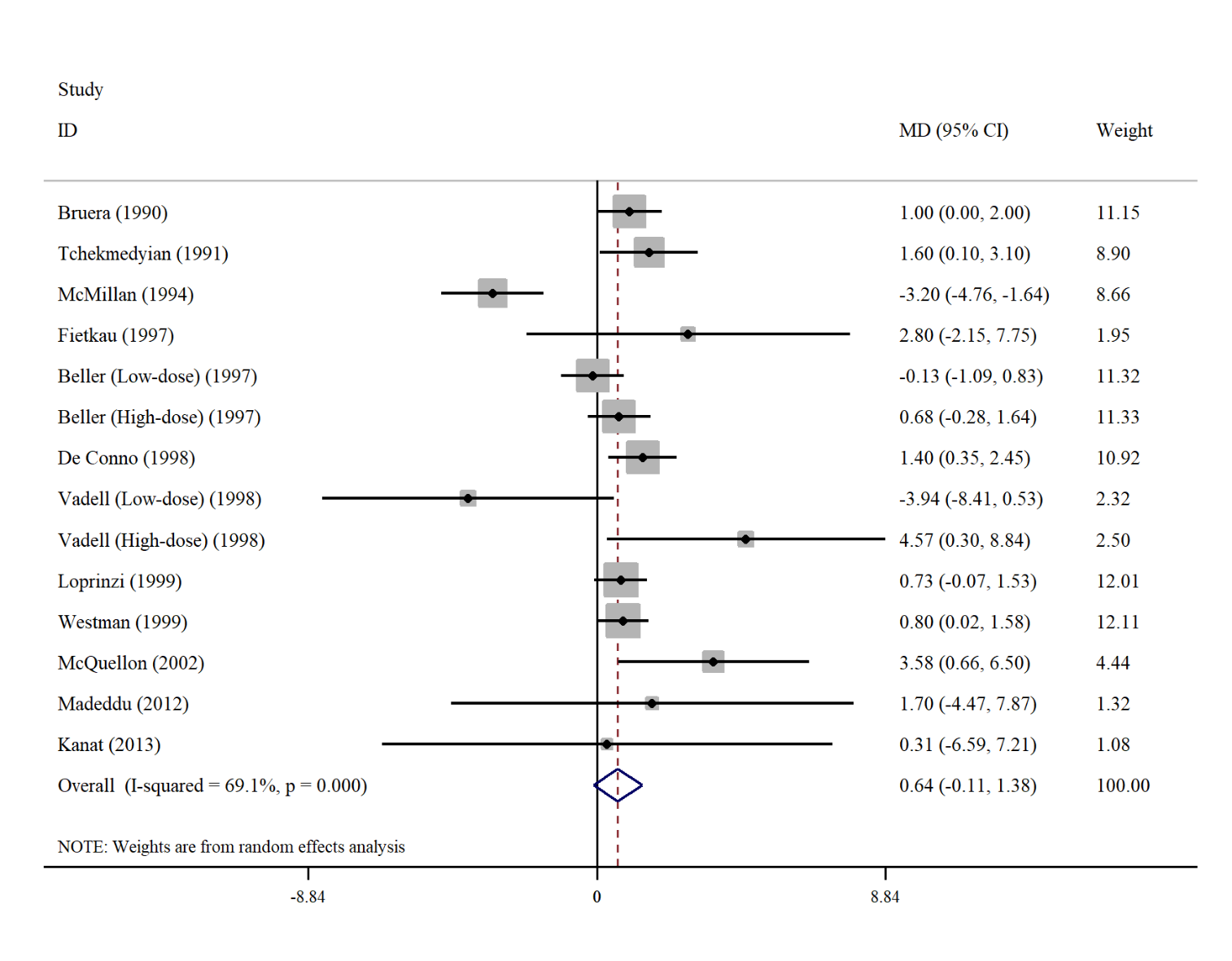


**Figure S1.** Forest plot of the effect of megestrol acetate supplementation on weight using random effects model. MD: mean difference, CI: confidence interval.


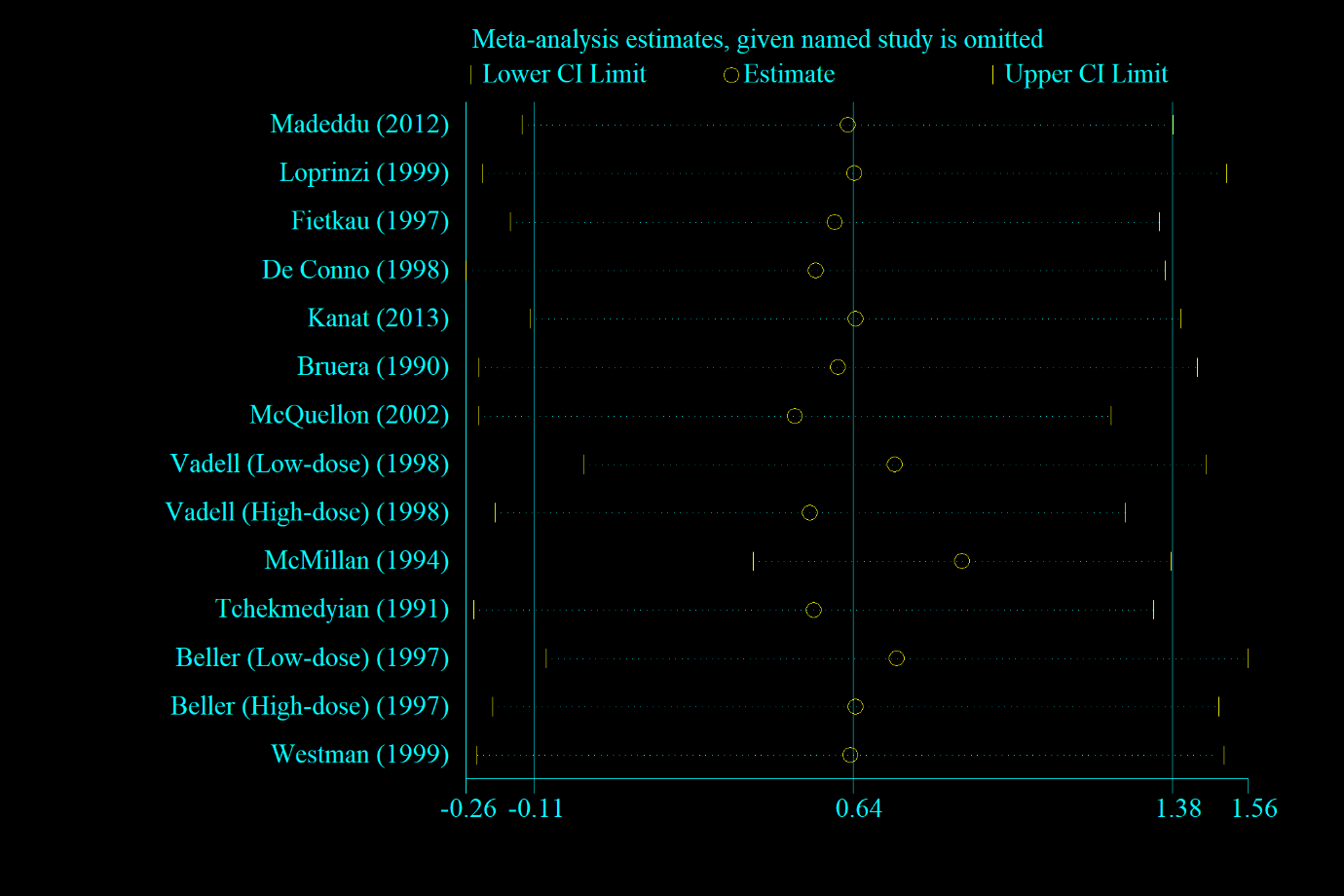


**Figure S2.** Forest plots show sensitivity analysis results of weight.


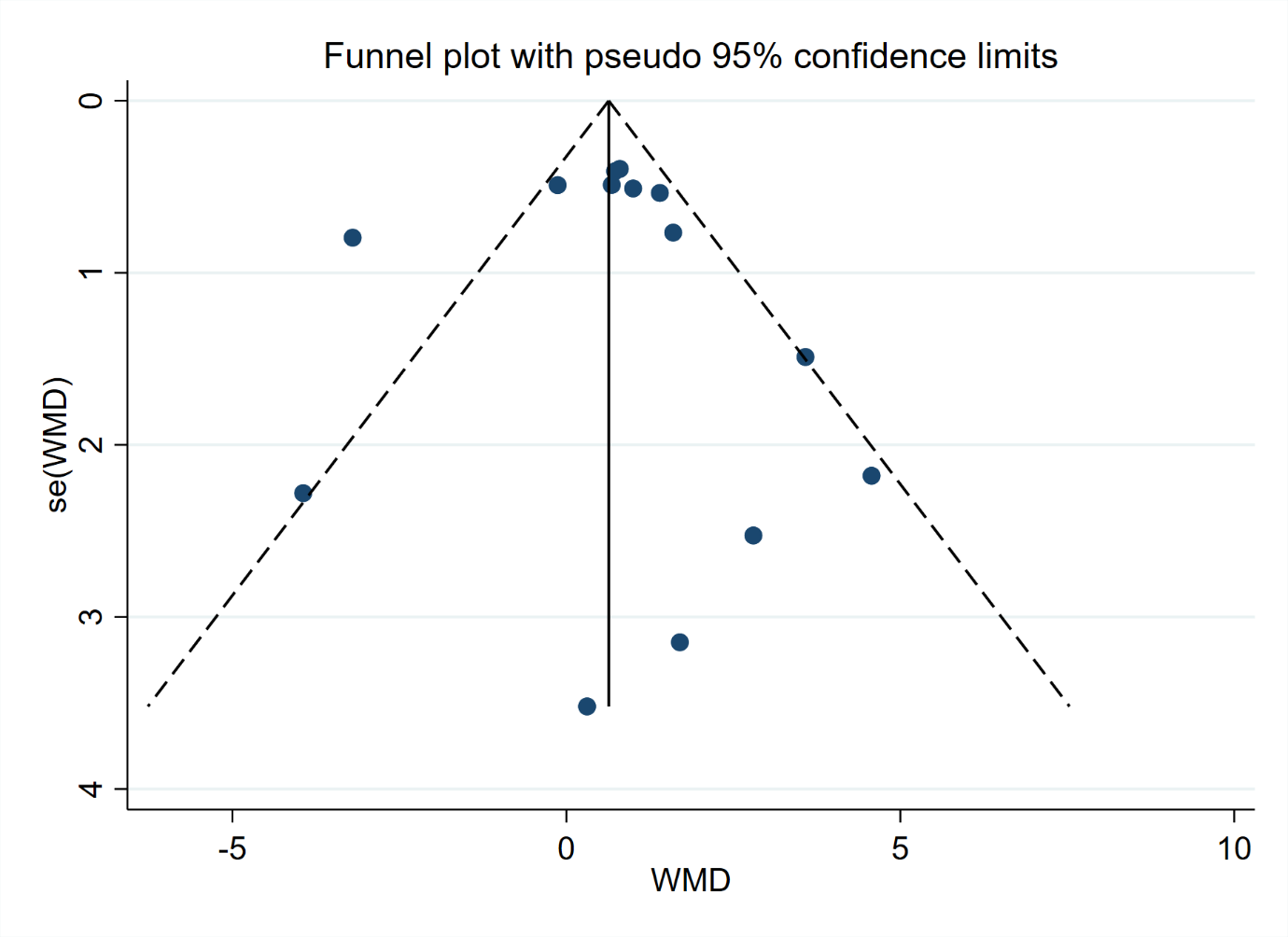


**Figure S3.** Funnel plot for evaluation publication bias of weight.


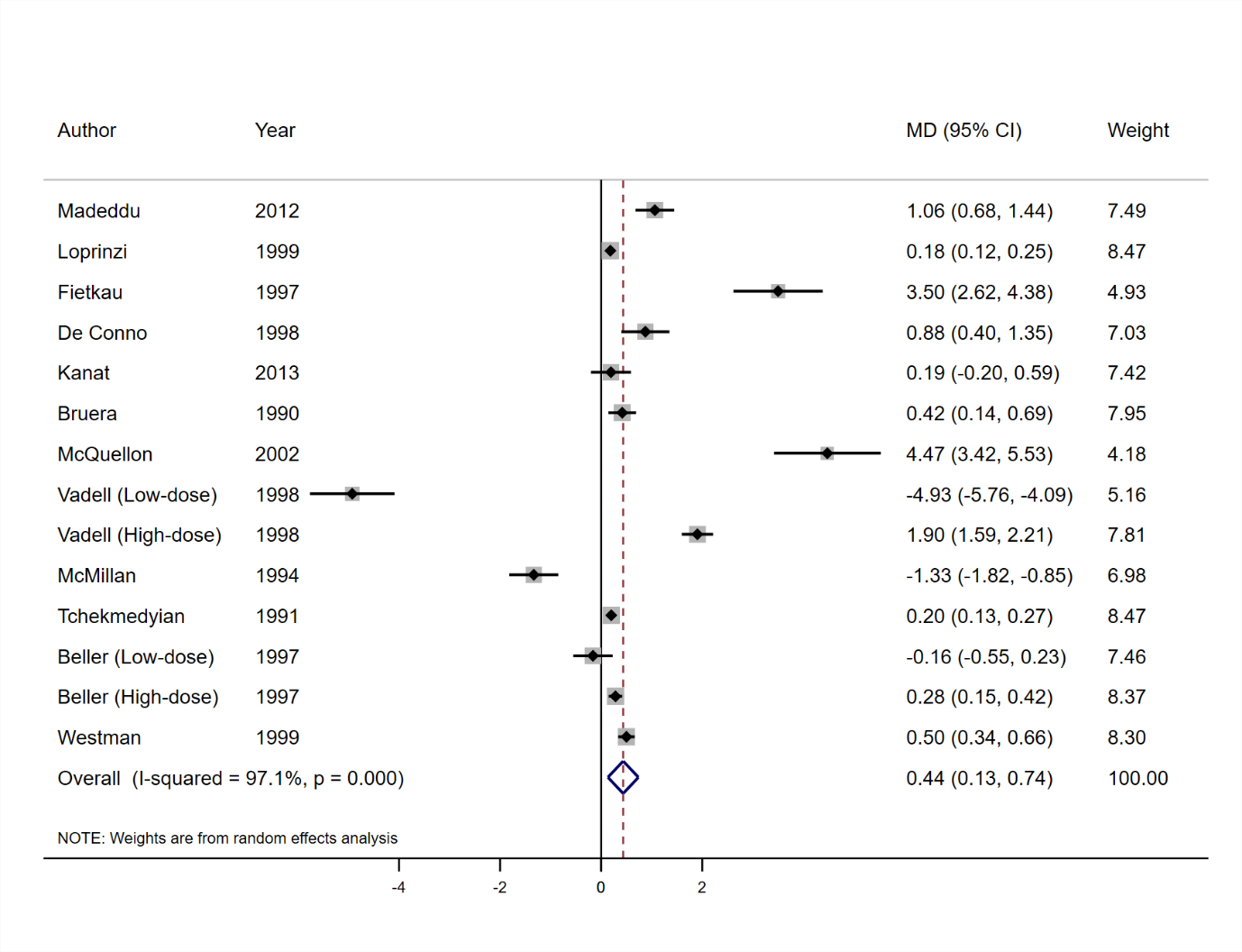


**Figure S4.** Weighted mean difference of weight for a 200 mg/d increment in megestrol acetate supplementation using random effects model. MD: mean difference, CI: confidence interval.


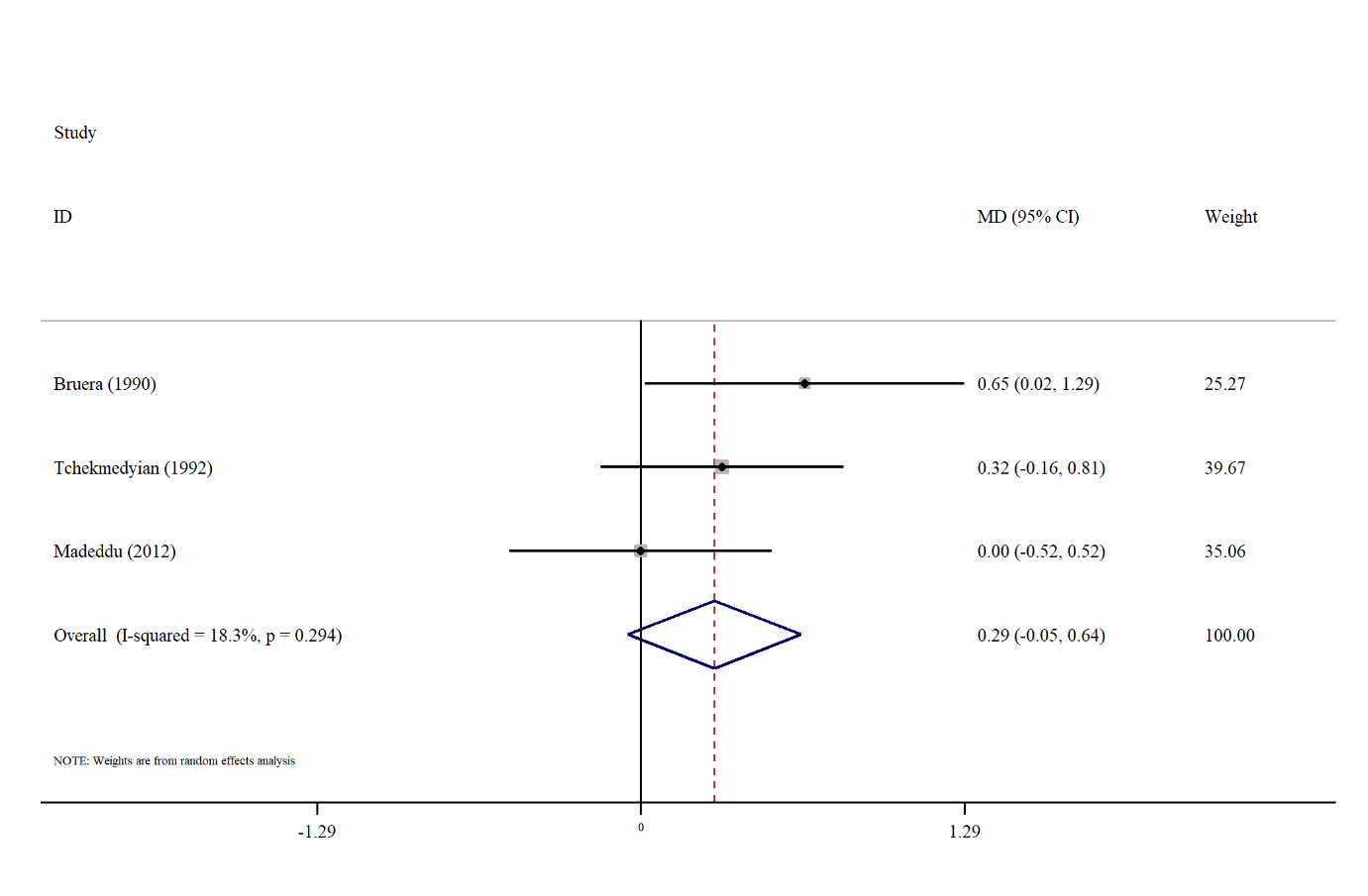


**Figure S5.** Forest plot of the effect of megestrol acetate supplementation on appetite using random effects model. MD: mean difference, CI: confidence interval.


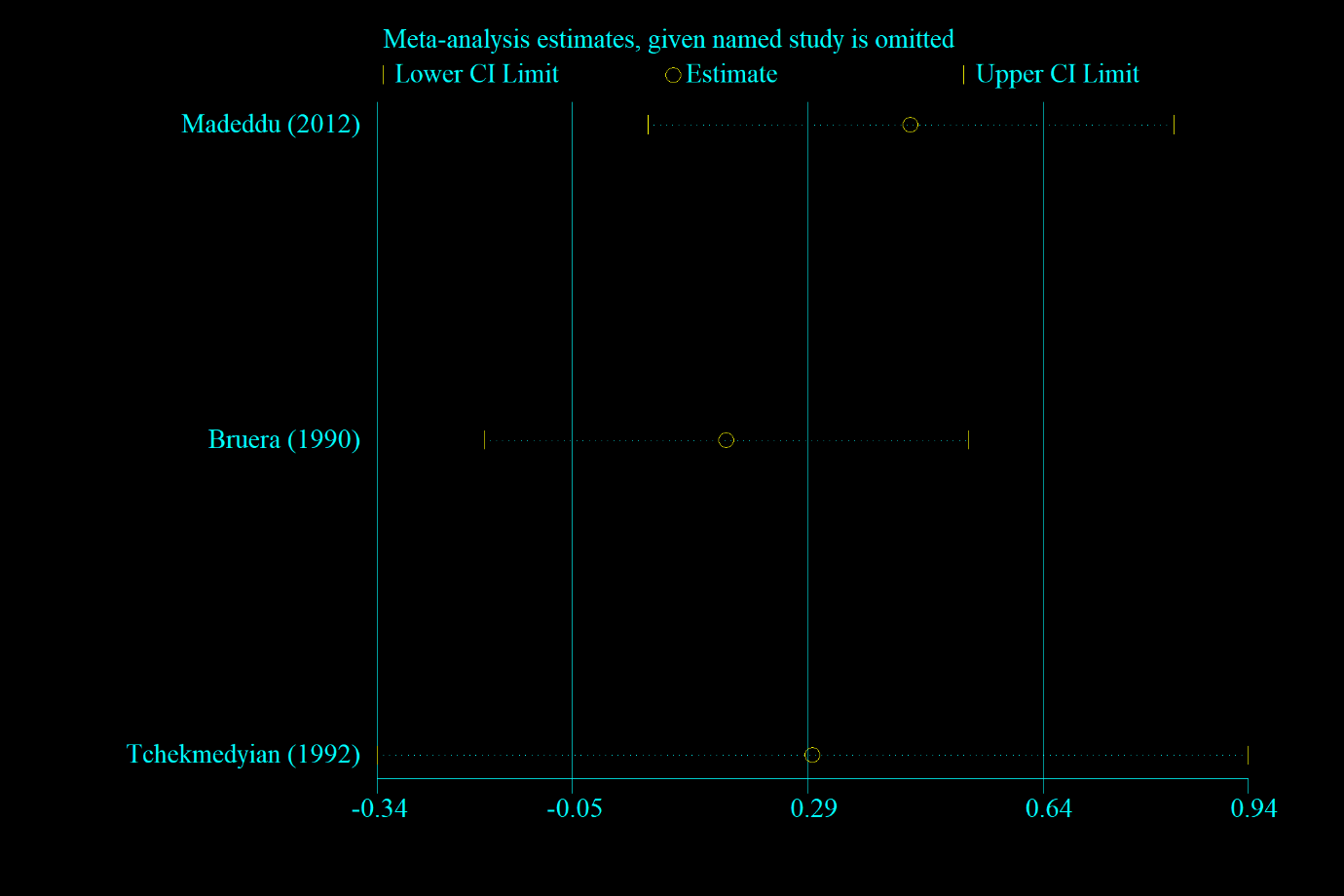


**Figure S6.** Forest plots show sensitivity analysis results of appetite.


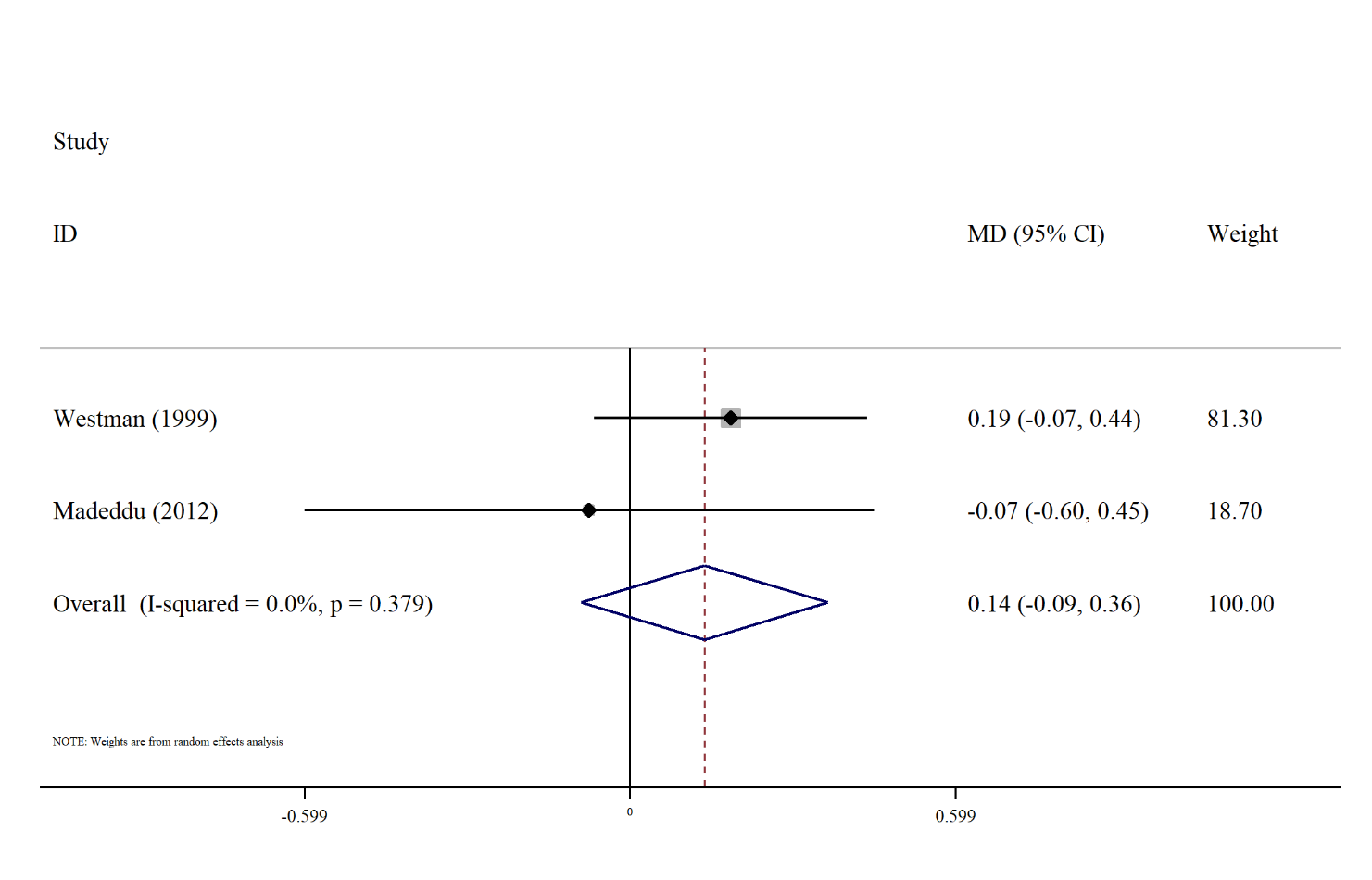


**Figure S7.** Forest plot of the effect of megestrol acetate supplementation on fatigue using random effects model. MD: mean difference, CI: confidence interval.


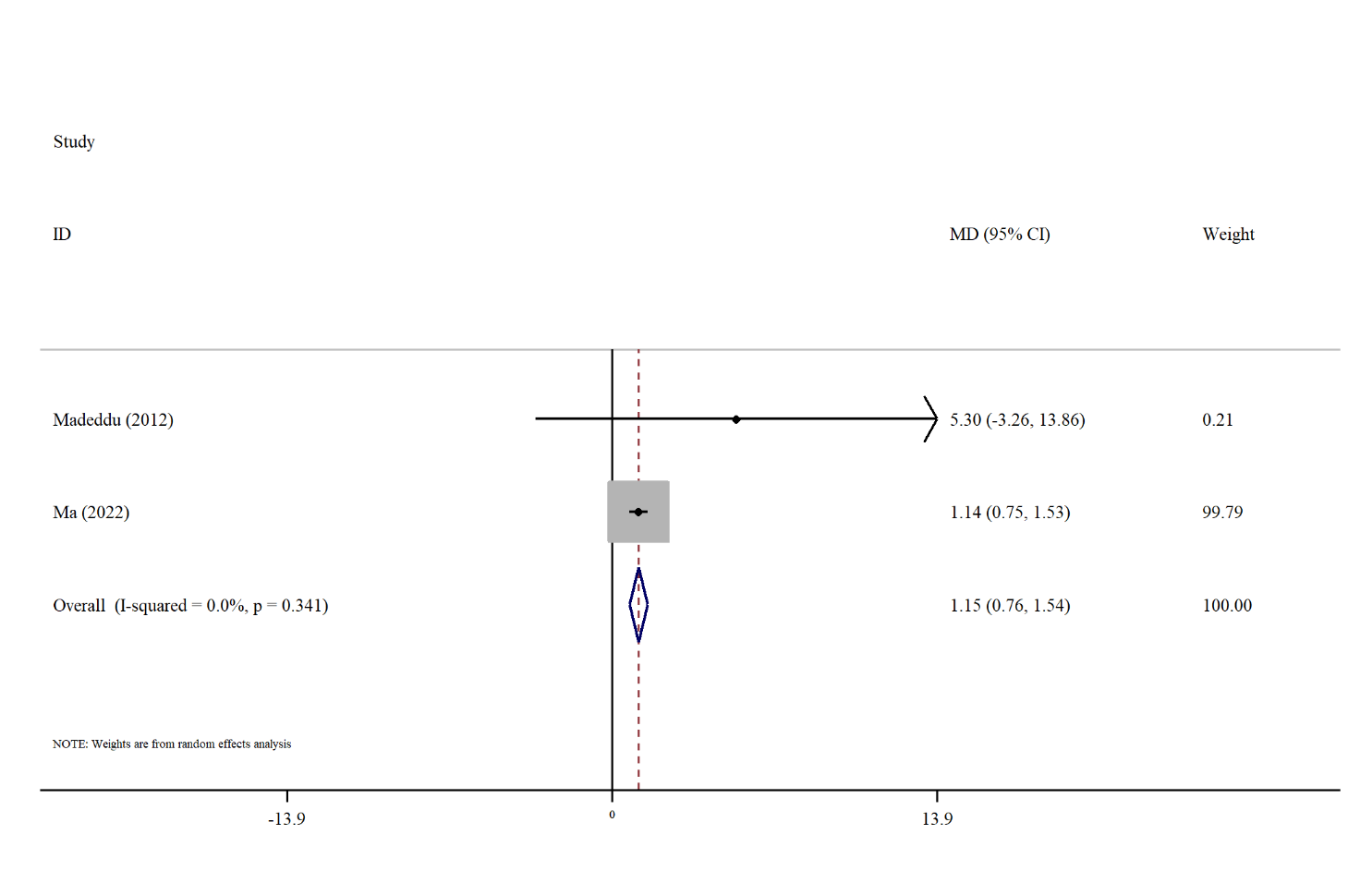


**Figure S8.** Forest plot of the effect of megestrol acetate supplementation on European Organization for Research and Treatment of Cancer Quality of Life Questionnaire C30 using random effects model. MD: mean difference, CI: confidence interval.
